# Age-of-acquisition affects object recognition and compound word identification: Evidence from visual duration thresholds and progressive demasking

**DOI:** 10.3758/s13414-026-03267-y

**Published:** 2026-05-13

**Authors:** Mahmoud M. Elsherif, Richard D. Kirkden, Jonathan Catling

**Affiliations:** 1 School of Psychology, University of Birmingham, Birmingham, UK; 2 School of Psychology and Vision Sciences, University of Leicester, 15 Lancaster Road, Leicester, LE1 7HA UK

**Keywords:** Age of acquisition, Compound word, Frequency, Progressive demasking task, Visual duration threshold

## Abstract

Items acquired during childhood are processed more quickly and accurately than those acquired during adulthood, a phenomenon known as the age of acquisition (AoA) effect. One explanation is that the AoA effect results from the arbitrary nature of the input–output mapping during the development of a lexical-semantic representation. However, previous behavioural studies have not directly compared the AoA effects in pictures and word processing. To investigate this, 48 British English students completed a visual duration threshold task and four variations of progressive demasking experiments. In these tasks, participants were shown 150 compound words, either unspaced or spaced, while attempting to identify the modifier, head, or the full compound word. In addition, in Experiment 4, we used a visual duration threshold task (VDT) for 150 objects that represented 150 compound words to assess the mapping theory. We observed that early-acquired items were identified more quickly than late-acquired items across all tasks, including the VDT. The AoA effect was strongest for a progressive demasking task (PDT) with unspaced and spaced compound words, followed by the VDT for pictures and PDT with component identification for spaced compound words. The only exception was the PDT with component identification of unspaced compound words, where the effect was not present. These results align with the integrated account, as the AoA effect is rooted in semantic representation during word identification but is also influenced by the connection between perception and semantics in the VDT.

**Supplementary Information:**

The online version contains supplementary material available at 10.3758/s13414-026-03267-y.

One predictor that contributes to the retrieval times for picture names, words and multiword units is the age-of-acquisition (AoA) of the item (e.g., Carroll & White, 1973). The AoA effect is the period within which a word, phrase or concept has been learned. It is a truism that items acquired earlier in life are easier and faster to retrieve than those acquired later in life and has been demonstrated across tasks (see review by Elsherif et al., 2023; Johnston & Barry, 2006; Juhasz, 2005). Although early-acquired items are more likely to be frequent and have more competitors than late-acquired items (e.g., Karimi & Diaz, 2020; Strain et al., 1995; Zevin & Seidenberg, 2004), it is difficult to reduce the AoA effect to these related variables (Brysbaert, 2017; Davies et al., 2017; see Juhasz et al., 2025, for evidence of reliability and validity of AoA ratings). Even though AoA cannot be reduced to these measures, both AoA and frequency show similar patterns within the linguistic system (Brysbaert & Ellis, 2016).

## Theories of AoA

### Representation theory

Two theories explain the AoA effect: representation theory, and network plasticity (Brysbaert & Ellis, 2016; Catling & Elsherif, 2020; Y. Chang & Lee, 2020; Y.-N. Chang et al., 2019). The representation theory argues that the AoA effect is the result of a lexical-semantic representation that develops over one’s lifespan (Brysbaert & Ghyselinck, 2006; Steyvers & Tenenbaum, 2005). Concepts acquired earlier during childhood have more semantic connections to other concepts than those acquired later in life, as the former are the centre of the semantic network with the latter being farther from the centre. Early-acquired concepts are thus more likely to be processed quicker, less likely to be forgotten and immune to cognitive deterioration (Catling et al., 2013; Henry & Kuperman, 2013; Marful et al., 2016; Steyvers & Tenenbaum, 2005). Evidence in favour of this account is from tasks that tap into semantic processing, such as semantic decision task (Brysbaert et al., 2000), and from tasks that are modulated by semantic variables (e.g., imageability, familiarity), such that the larger the semantic variable, the larger the AoA effect (e.g., Catling et al., 2021; Cortese et al., 2018; Elsherif et al., 2020; Sailor et al., 2011; Yum & Law, 2019).

### Mapping theory

The second theory of the AoA effect is the mapping theory of AoA (Ellis & Lambon Ralph, 2000; Lambon-Ralph & Ehsan, 2006; Monaghan & Ellis, 2002, 2010; Sohrabi, 2018; Zevin & Seidenberg, 2002, 2004). According to the mapping theory, the AoA effects are stronger when the relationship between an item's input and its output is less systematic. This is because the neural network is highly plastic during the acquisition of early items, allowing for the formation of robust and stable representations. As these items are learned, they modify the network’s connections and reduce its plasticity, making it more challenging to consolidate later-acquired items. This process gives early-acquired items an advantage in both recognition and production. The mapping theory argues that the AoA effect is not localized to a single processing system but rather emerges from the connections between different levels of representation, such as perceptual, semantic and phonological representations. This suggests the effect is distributed across the entire system rather than isolated to a single component (Y.-N. Chang et al., 2019). Supporting evidence includes picture-naming and word-naming tasks with the same stimuli (Catling & Elsherif, 2020; Lambon Ralph & Ehsan, 2006) wherein the AoA effects were found to be larger in picture naming than word naming tasks (pictorial stimuli have a more arbitrary link between perception, semantics and phonology than word stimuli). Research shows that the AoA effect is more pronounced when reading aloud words with inconsistent spelling-to-sound relationships (e.g., ‘yacht’) than a consistent spelling-to-sound relationship (e.g., ‘cat’) across different languages (e.g., Chinese: Y. Chang & Lee, 2020; Xue et al., 2017; English: Cortese & Schock, 2013; Italian: Wilson et al., 2012) because the connection between spelling and sound is more arbitrary in the former than the latter.

These theories are not mutually exclusive. An integrated perspective, which combines the representation account with mapping theory, can effectively explain AoA effects (Y.-N. Chang et al., 2019; Cortese et al., 2020; Dirix & Duyck, 2017a, 2017b; Elsherif, 2026; Elsherif & Catling, 2021, 2023, 2024, under review; Menenti & Burani, 2007; J. Wang et al., 2024). This integrated account posits that the mental lexicon's development is shaped by lifelong learning experiences (Brysbaert & Ellis, 2016; Y.-N. Chang et al., 2019; Elsherif et al., 2023). Words acquired early in life are processed more efficiently than late-acquired words because they form the lexicon's core. These early words have more connections within the neural network and have benefited from greater neural plasticity during development (Brysbaert & Ellis, 2016; Y.-N. Chang et al., 2019; Elsherif et al., 2023; Elsherif & Catling, 2024). Elsherif and Catling (2024) investigated the AoA effect using lexical decision tasks in different modalities (visual, auditory and cross-modal). They observed the strongest AoA effect in the visual task. Additionally, the authors noted that factors such as imageability, frequency, and familiarity—often considered measures of lexical-semantic processing (Juhasz, 2018; Juhasz et al., 2015)—influenced reaction times for compound words, a finding that supports the representation theory. Conversely, the mapping theory was supported by the auditory task, which showed a weaker AoA effect than the visual task, while the more consistent and regular cross-modal task showed no AoA effect. These findings suggest that both theories are needed to fully explain the AoA effect.

While the integrated account is a comprehensive explanation for the AoA effect, its origins may extend to even earlier cognitive processes, though research on these initial influences is limited. A growing body of evidence supports AoA effects on the earliest stages of visual recognition for both objects and words. For instance, Moore et al. (2004) found that early-acquired objects were classified more quickly in a "real or not real" judgment task that does not necessitate phonological or semantic access, suggesting the AoA effect may be a result of early visual identification (but see Elsherif, under review, who argued that semantic representations were, in fact, accessed). Similarly, Dent et al. (2007) reported a significantly lower visual duration threshold (VDT)—the minimum exposure time needed for correct object identification—for pictures with early-acquired names. This demonstrates that AoA affects the efficiency of visual object recognition by reducing the time required to acquire visual information. Catling et al. (2008) further found that both AoA and frequency contributed to the VDT. They also observed a larger AoA effect for degraded images (with irrelevant contours), which specifically affects perceptual processes. In contrast, the frequency effect remained similar across conditions (but see Preece, 2015). This suggests that the AoA effect arises at a preconceptual stage, while word frequency contributes at a post conceptual stage. The precise nature of this early influence, however, remains a subject of debate. Ploetz and Yates (2016) used a progressive demasking task (PDT)—a method that gradually reveals a word from a pattern mask to specifically assess early-stage lexical processing. They observed that imageability effects were larger for late-acquired words than for early-acquired words (see also Chen et al., 2009; Ghyselinck et al., 2004). Based on this, they concluded that while the AoA effect is driven by the orthographic-to-semantic connection, the imageability effect arises from semantic feedback to orthographic processing.

To address this question, we need to investigate the AoA effect with word and pictorial stimuli. The different cognitive mechanisms underlying object and word recognition lead to varying degrees of the AoA effect (see review by Elsherif et al., 2023). Object recognition relies more heavily on conceptual processing and involves a less direct mapping between perceptual, semantic, and phonological representations compared with word recognition. Catling and Elsherif (2020) supported this with their study where participants were presented with a prime (either a word or a picture) and a target (either a picture or a word) and asked to indicate if they matched. The authors observed that the AoA effect was similar in the picture–word verification and falsification tasks. However, the AoA effect was larger in the word–picture verification task than in the word–picture falsification task. They argued that the falsification task required access only to perceptual processes, while the verification task required access to conceptual representations. This subtle methodological difference resulted in larger AoA effects for object recognition than for word recognition (Catling & Elsherif, 2020; see review by Elsherif et al., 2023; Lambon Ralph & Ehsan, 2006). These findings suggest that the observed AoA effect may be partially influenced by semantic processing or the strength of connections between perceptual, semantic, and phonological representations.

Hitherto, the comparison of objects and words regarding the AoA effect has been primarily limited to monomorphemic words, which are less likely to involve semantic processing than compound words (e.g., 'seaplane'; Cortese & Schock, 2013; Elsherif et al., 2020). The majority of compound words, in English, are right-headed (Plag, 2018, Williams, 1981). Compound words, which are longer and less arbitrary, are more likely to depend on semantic processing to aid pronunciation (Elsherif, 2026; Elsherif & Catling, 2021, 2023, 2024; Elsherif et al., 2020; Juhasz, 2018; Juhasz et al., 2015; J. Wang et al., 2024). Common across many languages, compound words are typically formed by a head (e.g., 'plane’ in 'seaplane) and a modifier (e.g., 'sea’). The head’s morphological and syntactic features define the compound's semantic class. For example, if the head is a noun, the compound word is almost always a noun (e.g., ‘seaplane’ is a type of plane for the sea; Benczes, 2005, 2014, 2015; Günther et al., 2020). Because compound words have a less predictable orthographic–phonological correspondence, readers spend more time processing them, which allows their semantic properties to contribute more significantly to recognition (Elsherif et al., 2020).

English compound words, unlike many other languages, exhibit varied spatial layouts in their written form: open (e.g., 'sweet corn’), hyphenated (e.g., sweet-corn) and closed (e.g., ‘sweetcorn'). This categorization is not always consistent, as different dictionaries may classify the same compound word differently (Juhasz et al., 2005; Kuperman & Bertram, 2013). Research indicates that this spatial layout can interact with other lexical properties, such as the morphemic salience (how easily the component parts are recognized) and the semantic relationship between them (Bertram, 2011; Elsherif et al., 2020; Elsherif & Catling, 2023, 2024; Inhoff et al., 2000). For instance, adding spaces to German compounds, which is grammatically incorrect, has been shown to improve processing speed compared with their unspaced forms (Inhoff et al., 2000). However, the effects of spacing in English compounds appear less consistent. Elsherif et al. (2020) found no effect on naming latencies, while Elsherif and Catling (2023) reported slower decision times for spaced compounds compared with unspaced ones in a memory task (but see Elsherif & Catling, 2024, who showed spaced compounds responded more quickly than unspaced compound words). Given their established status as lexicalized word forms in dictionaries and their integration into the mental lexicon of English speakers, this study focuses specifically on unspaced and spaced compound words.

However, there are a few studies conducted on the AoA effect in compound word recognition (e.g., Bonin et al., 2022; Elsherif & Catling, 2021, 2023, 2024; Elsherif et al., 2020; Juhasz, 2018; Juhasz et al., 2015). Juhasz et al. (2015) noted that the AoA of compound words, not of individual morphemes, affected lexical decision task and naming latencies. The authors argued that the AoA effect occurs at the lexical-semantic level, as the individual morphemes are not activated obligatorily when compound words are processed (see Juhasz, 2018, who replicated the findings in eye-tracking; Kuperman & Bertram, 2013, who noted that imageability and concreteness of the compound word, not the individual morphemes, contributed to the latencies). Nevertheless, morphemic decomposition occurs, as the frequency, AoA, imageability or familiarity of the modifier and head lexeme influences compound word recognition (Elsherif et al., 2020; Elsherif & Catling, 2021, 2023, 2024; Juhasz et al., 2003). Elsherif et al. (2020) explored how the AoA effect influenced the processing of compound words and noted that the AoA of the compound word contributed to the processing of compound words in both unspaced (e.g., ‘sunflower’) and spaced (e.g., ‘sun flower’) compound words. However, the difference in compound word presentation can lead to the presence of the AoA of the modifier and head lexeme in word naming such that the earlier the head or modifier lexeme is acquired, the faster the naming latencies (see also Elsherif & Catling, 2024, who replicated these findings in a lexical decision task). Overall, the research provides clear evidence for the AoA effect in compound words, during naming and semantic tasks but not the earlier processes. While morphemic decomposition seems to influence processing speed in spaced compound words, further research is needed to fully understand how the AoA of a compound word’s individual parts interacts with the processing of the compound word as a whole. This may only occur during the earlier parts of the identification of the compound word.

To summarise, this research addresses the above knowledge gaps to examine the AoA effects in compound words and objects that instantiate their label. Specifically, it investigates how the AoA effect manifests in compound words, using two methods: progressive demasking and visual duration threshold with pictures. This approach will provide a more comprehensive understanding of the factors that influence the AoA effect in the early stages of word recognition and object recognition. Ultimately, the goal is to determine how these factors align with the integrated account of the AoA effect and whether the AoA effect begins earlier than previously thought, possible even at the perceptual stage.

The predictions for the five experiments were straightforward, based on previous findings and theoretical frameworks. In Experiment 1 (unspaced PDT), consistent with prior research (e.g., Ploetz & Yates, 2016; Preece, 2015), AoA was predicted to influence performance. Words acquired earlier in life were expected to be identified with a smaller stimulus duration, as earlier-learned words are generally processed more efficiently. In Experiment 2 (spaced progressive demasking), both AoA and frequency were predicted to affect performance. The key prediction was that adding a space between morphemes would make the AoA effect more pronounced. This is because the AoA effect is closely tied to the ease of accessing a word’s meaning. By separating the morphemes, the initial meaning of the individual parts might become more readily available, potentially amplifying the influence of AoA on the time it takes to recognize the entire compound word. Consequently, the AoA of both the modifier and head lexeme were predicted to influence performance. The PDT requires rapid and efficient mapping from a partial visual input to a full-fledged orthographic, phonological, and semantic representation (Catling et al., 2008), while the spaced PDT, by disrupting the standard orthographic form of the compound, would specifically tax these mappings (Elsherif et al., 2023, 2024). In line with the mapping theory, we predict that the AoA effect is more pronounced in spaced than unspaced compound words, as early-acquired words have strong internal connections that might be more resilient to this disruption, thus would be more easily accessed than that of late-acquired compound words.

In Experiment 3a (unspaced progressive demasking with component identification), building on Elsherif and Catling (2021), the focus was on identifying a single component of a compound word while the other was held constant. AoA was predicted to contribute to the task, with words acquired earlier in life requiring a smaller stimulus duration for identification. However, the effect was expected to be small because the task, which focuses on a single lexeme, was considered easier, leading to faster recognition times. The task is more systematic and regular, as it focuses on the monomorphemic word more than the compound word itself. The same experiment is repeated in Experiment 3b (spaced progressive demasking with component identification). The prediction was that adding spaces would make the AoA effect more pronounced, as it would require linking between the two morphemes. It was unclear whether this experiment would take more or less time than Experiment 3. While the task involves simpler monomorphemic words, the difference in latencies might not be significant. This is because separating the morphemes was expected to increase reliance on deliberate processing, amplifying the influence of AoA and frequency on the time taken to identify both the head and modifier lexemes. According to the mapping theory, as these tasks rely on mapping from a visual form to a meaning on a morphemic level, the effect would be driven by the strength of the mapping to the monomorphemic word to the compound word itself. Early-acquired components would be identified faster and the effect would be smaller in the component identification tasks than that of Experiments 1 and 2.

Finally, in Experiment 4, building on Dent et al. (2007), we used a VDT with compound words to investigate whether the label of a compound word, and/or its individual morphemes, would contribute to the recognition of objects when it occurs during stimulus identification prior to recognition, counter to the findings of a previous study (Elsherif et al., under review). The use of VDT was designed to capture early processing effects and determine if the lack of morphemic effects observed in other studies was due to these effects being processed too quickly to be measured. The primary prediction was that compound words would be identified at a shorter duration, suggesting that early recognition processes may not require full morphemic decomposition. The VDT requires the time to establish the connection between the visual input and the conceptual representation. In line with the mapping theory, the more established and efficient mappings of early-acquired words would allow this process to happen faster, even under degraded visual conditions. We predict that the AoA effect should be similar across PDT and VDT.

In addition, according to the representation theory, the AoA effect should be largest in VDT followed by spaced PDT, as the former requires more access to the conceptual knowledge at the conceptual, morphemic (i.e., modifier and head), whole word and relational (i.e., the meaningful connections between the morphemes in a compound word; e.g., Lyons, 1963), while the spaced PDT requires access to morphemic, whole word and relational. This would be followed by unspaced PDT and spaced component identification as the former requires access to the whole word and the spaced component identification requires access to the morphemic and relational components. According to the mapping theory, the AoA effect is expected to be largest in VDT and spaced PDT, as the mapping between representations is more irregular, leading to a larger processing cost for late-acquired words. The unspaced PDT and component identification should show similar levels of AoA effect, as the process focuses primarily on the mapping between orthographic–semantic processing. However, the unspaced component identification should show no AoA effect, as the mapping between orthography and phonology is regular.

## Experiment 1: Unspaced progressive demasking

### Method

#### Data availability

The data and analyses are located via the Open Science Framework (https://osf.io/knwur/).

#### Sample-size justification

Using the formula for effect-size calculation from Westfall et al. (2014), we performed a power analysis on the findings of Elsherif et al. (2020). According to the power analysis, a minimum of 13 participants was required to achieve adequate power (*β* = 0.80). To ensure a sufficient sample size and reduce the risk of false positives, our study recruited 48 participants for each experiment, which was more than the required minimum^1^. Given the number of words in each task, there were 7,200 observations per task, which is above the recommendation of Brysbaert and Stevens (2018).

#### Participants

The participants in this study were 48 British undergraduates at the University of Leicester. The students were between 18 and 21 years of age (*M*_age_ = 18.77 ± 0.74 years; 30 women). All participants were monolingual, had normal or corrected-to-normal vision and had signed a consent form to participate in the study. The study was approved by the ethics committee of the University of Leicester (1574), and the participants received course credits for their participation.

#### Materials

We used the same stimuli as Elsherif (under review), which we briefly describe in this section. Each participant saw 150 words that were primarily noun–noun compounds (see the Appendix). All 150 compound words used in this study were highly concrete noun–noun compound words, as they were required to be representable as distinct physical objects from Elsherif (under review) and for Experiment 4 in the VDT. Word frequencies as Zipf values were extracted from the SUBTLEX-UK database for the compound word and modifier and head lexemes of the compound word (van Heuven et al., 2014). Letter length, imageability (measuring how easily a mental image is aroused by a word), AoA, semantic transparency (ST; i.e., the meaning of the compound word is related to its parts to a greater or lesser degree, falling somewhere on a continuous scale between a strong relation to the modifier and a strong relation to the head), and lexeme meaning dominance (LMD; i.e., the degree to which the meaning of a compound word is contained in either the head or the modifier) were taken from Elsherif’s (under review) collection of the norms. The name agreement, familiarity of the compound word, image agreement (how closely a picture resembles the mental image of the object) and visual complexity (the amount of detail or intricacy in the picture) for these pictorial stimuli were sourced from Janssen et al. (2011).The familiarity of the head and modifier lexeme were taken from their respective databases (familiarity; Balota et al., 2001; see Table 1 for psycholinguistic characteristics). We included name agreement and image agreement as critical control variables to ensure the internal validity of our cross-modal comparison and compare the cognitive mechanisms underlying word processing and object processing. These are two important predictors especially in the context of object recognition and picture naming (Alario et al., 2004; see the meta-analysis by Perret & Bonin, 2019). By including these factors, we were able to partial out the variance attributed to the visual and lexical properties of the stimuli themselves. This allowed us to isolate the specific effects of our target variables (e.g., AoA) without them being confounded by how easily the objects were recognized or named.

Although it is counterintuitive to include phonetic complexity as a measure of progressive demasking and visual duration threshold, Ferrand et al. (2011) observed that initial phonetic complexity is a strong predictor of progressive demasking threshold performance such that the first phoneme (or letter) has a surprisingly strong effect on progressive demasking (PDM)—accounting for roughly 42% of the variance. Because PDM is uniquely sensitive to these physical onset characteristics, we treated phonetic complexity as a control in our baseline model. As a result, we coded each word dichotomously (1 or −1) whether there was a presence of the feature (1) and absence of the feature (−1) in the following properties: bilabial, labiodental, dental, labiovelar, postalveolar, alveolar, palatal, glottal, velar and voiced. We did not select objects based on the above criteria but instead used a regression design, allowing the words to vary along dimensions of interest (e.g., semantic transparency; see Table 1).

**Table 1:** Compound word stimulus characteristics for 150 words in the analyses (standard deviations are in parentheses). (Seven words (‘oxcart’, ‘prizefight’, ‘turtledove’, ‘castoff’, ‘carryall’, ‘filmstrip’ and ‘campground’) were not located in SUBTLEX-UK, thus van Heuven et al. (2014) recommended to give items not found in this database a value of 0.696 to word frequency.)

Predictors	Compound word		Modifier		Head	
	*M* (*SD*)	Range	*M* (*SD*)	Range	*M* (*SD*)	Range
Orthographic length	8.98 (1.71)	6–15				
Frequency (out of 7)	2.39 (1.21)	0.696–5.12	4.63 (0.65)	2.92–6.43	4.47(0.73)	1.81–6.56
Familiarity (out of 7)	3.69 (1.35)	1.32–6.87	3.89 (1.90)	1–6.72	3.90(1.71)	1–6.72
Imageability (out of 7)	5.46 (0.68)	3.18–6.42	5.83 (1.02)	2.22–6.90	5.88(0.81)	2.9–6.9
AoA (out of 7)	3.86 (0.85)	2.32–5.71	3.09 (0.74)	2.10–5.48	3.33(0.65)	2–5.3
ST (out of 7)	4.74 (1.19)	1.74–6.31				
LMD (out of 10)	5.68 (0.84)	2.68–7.15				
Name agreement (H-index)	0.95 (0.80)	0–3.23				
Name Agreement (%)	67.53 (29.04)	0–100				
Visual Complexity (out of 7)	3.804 (1.303)	1.19–6.91				
Image agreement (out of 7)	5.10 (0.83)	2.39–6.78				

One discrepancy arose in the SUBTLEX-UK database (van Heuven et al., 2014), where the Zipf scale, which typically ranges up to a score of 7, was exceeded by some function words. For example, the word 'to' had a Zipf scale value of 7.42. This occurs because the Zipf scale is a logarithmic transformation of raw word frequency; while it compresses most data into the 1–7 range, extremely common words like 'to' are so frequent that their scores naturally fall above this typical maximum.

### Procedure

The experiment was preregistered on the Open Science Framework (https://osf.io/gzvyr). The study took place in a quiet room, with a participant individually completing a demographic questionnaire before beginning. The stimuli were presented on a 19-in. AG Neovo F-419 using Gorilla.sc software (Anwyl-Irvine et al., 2020a, b).

To ensure a stable refresh rate of 60 Hz, the study began with a frames per second (FPS) counter. Once successful, the experiment began. Each trial started with a 250-ms fixation cross. This was followed by a series of alternating pattern masks and unspaced compound words. The mask and the compound word alternated 19 times, with each alternation (referred to henceforth as a cycle) lasting 333.33 ms. The first cycle began with a 316.67 ms pattern mask composed of hash characters that matched the word's length, followed by the word presented in lowercase for 16.67 ms. A monospace font (Consolas) was used. In each subsequent cycle, the word’s duration increased by 16.67 ms, while the pattern mask’s duration decreased by the same amount. In the last cycle, the mask was presented for 16.67 ms and the word for 316.67 ms. Participants pressed the down arrow key when they believed they had identified the word. They were then prompted to type the word. After typing, they pressed the space bar to start the next trial. The order of words was randomized for each participant.

### Analyses

We preregistered the study plan, including the methods, analyses, outlier rejection, missing data and inference at the Open Science Framework (https://osf.io/gzvyr). All analyses were conducted in R statistical software (Version 3.6.1.; R Core Team, 2019), relying specifically on packages such as *tidyverse* (Version 1.3.0; Wickham et al., 2019) for data processing and *lme4* (Version 1.1.26; Bates, 2010) for data analysis and using linear mixed models, via the ‘lmer’ function.

The analyses focused on stimulus duration, defined as the duration of the compound word in the last cycle in which the participant viewed it before pressing the down arrow, for identifying the word. The stimulus duration was a continuous measure. In VDT and PDT, this threshold measure is prioritized over total reaction time (RT) as it specifically captures the efficiency of the initial mapping between sensory input and conceptual representation, minimising the influence of motor response variance (Catling et al., 2008; Dent et al., 2007). We acknowledge that brief stimulus durations on digital displays require careful psychophysical consideration regarding refresh rates and luminance signals (Elze, 2010a, 2010b). However, for the purposes of isolating lexical-semantic effects, the identification threshold remains the most theoretically valid measure. This is supported by our secondary analyses on total RT (see Supplementary Materials). Total RT (the interval from stimulus onset to participant response; see Supplementary Materials for RT) is a common metric in other paradigms. Secondary analyses were performed on Total RTs which yielded results that were not consistent with the primary duration analysis but in line with offset data (Scaltritti et al., 2016). The model variance being dominated exclusively by word length and phonetic complexity. This indicates that total RT primarily captures the postlexical planning and motor output processes, which are sensitive to physical size and articulatory difficulty. Consequently, following the precedent of Dent et al. (2007) and Catling et al. (2008), stimulus duration was used to ensure that the observed AoA effects reflect the quality of the lexical representation rather than the speed of the motor response.

To analyse this data on stimulus duration, we used linear mixed models (LMMs) with predictor variables entered as fixed values. We began with a maximal model, which included random subject and items intercepts, as well as random subject slopes. However, as maximal models rarely converge (Winter, 2019), we simplify the random effect structure, starting with the removal of random slopes and, if necessary, moving toward an intercept-only random-effects structure (Matuschek et al., 2017). Random slopes were systematically removed to resolve singular fits, ensuring that fixed-effect estimates remained mathematically stable and were not derived from overparameterised models. We did not transform the stimulus duration. To ensure the models were valid, we checked their assumptions based on Meteyard and Davies (2020) recommendations. The study also reported two types of R-squared values, marginal (*R*^2^m) and conditional (*R*^2^c), using the *MuMIn* package (Version 1.43.17; Barton & Barton, 2015; Nakagawa et al., 2013). The marginal R2 shows the variance explained by the fixed effects (the main variables of interest), while the conditional *R*^2^ includes the variance explained by both fixed and random effects (like individual participants or items).

To account for the high sensitivity of the demasking paradigm to initial phonetic and physical properties (Ferrand et al., 2011), we initially analysed the data using linear mixed-effects models (LMMs) that included a comprehensive set of phonetic complexity measures (e.g., place of articulation and voicing). However, following the principle of model parsimony and to ensure stable regression estimates (Achen, 2005), nonsignificant phonological predictors were pruned from the final models reported here. Our final baseline models follow the benchmark methodology of Kuperman (2013) and Juhasz (2018), accounting for variance attributed to whole compound word frequency (log-transformed), compound length, and the frequencies of the constituent lexemes (modifier and head)^2^. In accordance with previous studies (e.g., Elsherif, under review; Elsherif & Catling, 2021, 2023, 2024; under review; Elsherif et al., 2020; Juhasz, 2018; Juhasz et al., 2015; Kuperman, 2013), each theoretical variable of interest, specifically the AoA of the compound and its constituents, were subsequently added to this baseline to test for incremental explanatory power. This hierarchical structure ensures that the reported lexical-semantic effects are robust to the perceptual variance that typically explains a large portion of the *R*^2^ in progressive demasking tasks.

**Fig. 1:**
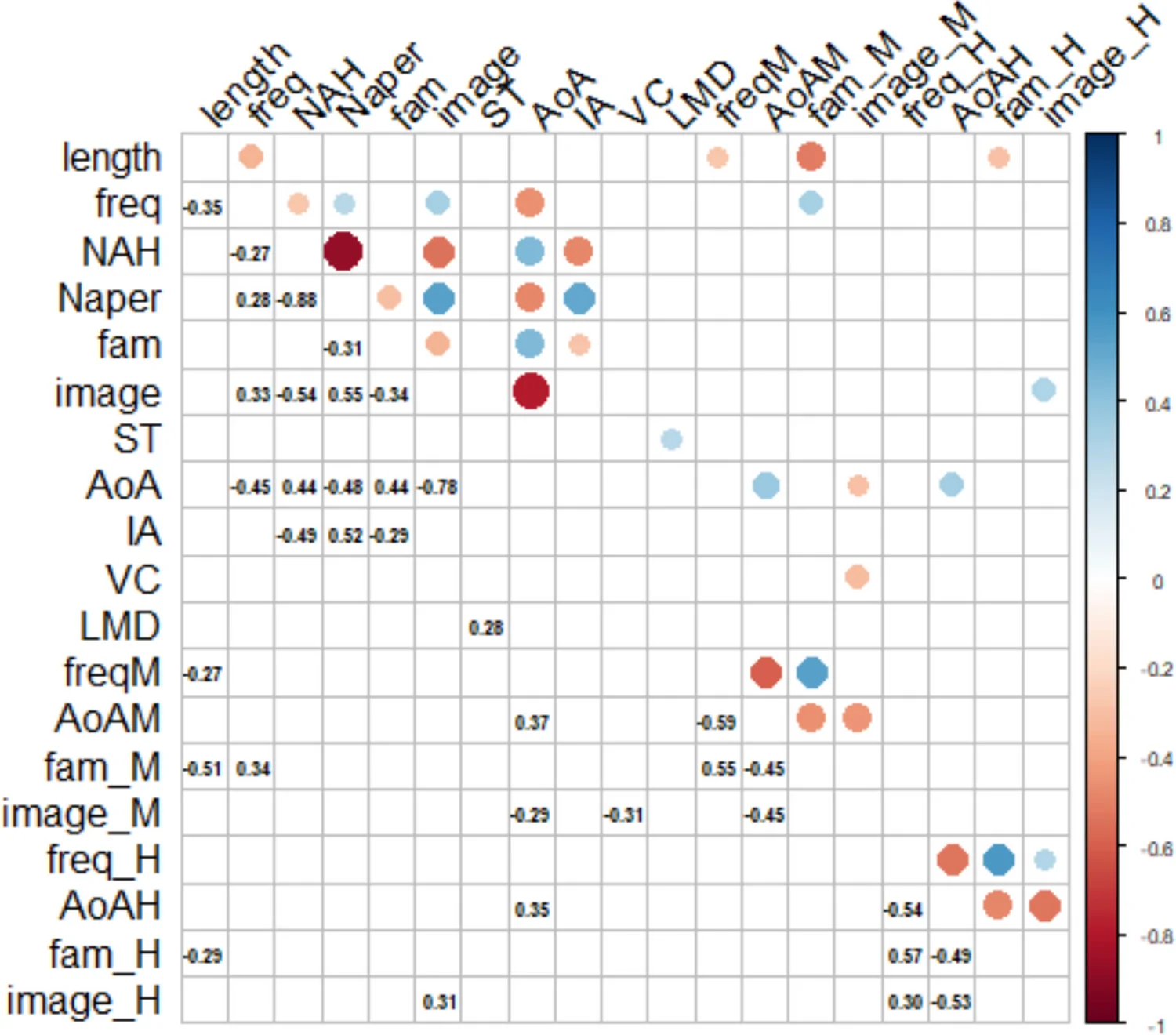
Pearson correlations between all item properties and outcome measures for 150 words. The strengths and directions of coefficients are indicated by the size and colour of circles above the diagonal, and the correlation coefficients are reported below the diagonal. Only correlations significant at *p* < .001 are shown, and any correlations with a *p*-value greater than .001 have been suppressed. Length = letter length, freq = word frequency, NAH = name agreement (H); Naper = name agreement (%); fam = concept familiarity; image = imageability; ST = semantic transparency; AoA = age of acquisition; IA = image agreement; VC = visual complexity; LMD = lexeme meaning dominance; freqM = frequency of the modifier; AoAM = age of acquisition of the modifier lexeme; fam_M = familiarity of the modifier; image_M = imageability of the modifier; freq_H = frequency of the head lexeme, AoA_H = AoA of the head lexeme; fam_H = familiarity of the head lexeme; image_H = imageability of head lexeme. (Colour figure online)

To ensure the statistical integrity of our models, all continuous predictors were centred on their means. Multicollinearity was assessed using the variance inflation factor (VIF); across all models, VIF values remained low (approximately 1.09), well below the traditional threshold of 5. The random effects structure initially included by-participant and by-item intercepts, as well as by-participant random slopes for the predictors. However, in cases of nonconvergence or singular fits, the random effect structure was simplified following the parsimonious modelling approach advocated by Matuschek et al. (2017). To isolate the unique contribution of each theoretical factor while controlling for shared variance, predictors were added to the baseline model individually. This incremental modelling approach, combined with a low mean VIF (1.09), ensured that the effects of AoA and morphemic structure were not suppressed or inflated by multicollinearity between the predictors (see Fig. 1 for the correlation matrix). This approach follows the hierarchical modelling logic of Kuperman (2013), isolating the specific contribution of mapping-related variables from general lexical factors. To maintain a conservative family-wise error rate across our planned comparisons, all *p*-values were adjusted using the Holm–Bonferroni method.

## Results

### Unspaced PDT

Following the advice of Woods et al. (2023), the rate of missingness (i.e., lacking a data point from the participant or item) across the samples was less than 1% and was considered missing at random. The average stimulus duration was 79.1 ms (*SD* = 37.4, 95% CI [78.2, 80]). We investigated the impact of word frequency, AoA, familiarity, semantic transparency, imageability, lexeme meaning dominance (LMD) and morphemic properties on stimulus duration for unspaced PDT.

In the baseline model, we observed a significant effect of the compound word frequency and head frequency on stimulus duration rates: high-frequency compound words took less time to identify than low-frequency ones (Table 2). High-frequency head lexemes took less time to identify than low-frequency ones. In the baseline + predictors model, AoA of the compound word influenced stimulus duration, as compounds acquired earlier in life were identified faster. Name agreement (both H index and percentage) also significantly influenced stimulus duration (Table 3). Unspaced compound words with higher name agreement and less spread took less time to identify. Furthermore, familiarity and imageability of the compound word were significant predictors, with higher familiarity and imageability leading to shorter identification times. Furthermore, the AoA of the modifier also significantly influenced stimulus duration, such that compounds with a modifier acquired earlier in life were identified faster.

**Table 2:** The baseline model results for stimulus duration of correct responses in the progressive demasking task*

Effect			Stimulus duration				
	*β*	*SE*	2.5%	97.5%	*T*	*R*^2^*m*	*R*^2^*c*
*Experiment 1: Unspaced compound word PDT*^*a*^							
L	0.065	1.239	−2.363	2.493	0.53	0.069	0.412
CFreq	**−8.999**	**1.242**	**−11.43**	**−6.565**	−7.25*	**0.069**	**0.412**
MFreq	0.745	1.189	−1.585	3.075	−0.63	0.069	0.412
HFreq	**−2.443**	**1.162**	**−4.720**	**−0.165**	**−2.10**	**0.069**	**0.412**
*Experiment 2: Spaced compound word PDT*^*a*^							
L	0.008	0.696	−1.356	1.372	0.12	0.003	0.507
CFreq	**−1.614**	**0.698**	**−2.982**	**−0.246**	−2.31*	**0.003**	**0.507**
MFreq	−0.002	0.668	−1.311	1.307	−0.00	0.003	0.507
HFreq	−0.658	0.654	−1.94	0.624	−1.01	0.003	0.507
*Experiment 3a: Unspaced compound word—Identification of the Modifier lexeme *^*a*^							
L	−0.140	0.355	−0.836	0.556	−0.39	0.0006	0.409
CFreq	0.681	0.352	−0.009	1.371	1.94	0.0006	0.409
MFreq	−0.214	0.341	−0.882	0.454	−0.62	0.0006	0.409
HFreq	−0.645	0.336	−1.304	0.014	−1.92	0.0006	0.409
*Experiment 3a: Unspaced compound word—Identification of the Head lexeme *^*a*^							
L	0.177	0.368	−0.544	0.898	0.48	0.0003	0.373
CFreq	−0.026	0.372	−0.755	0.703	−0.07	0.0003	0.373
MFreq	−0.469	0.354	−1.163	0.225	1.32	0.0003	0.373
HFreq	0.165	0.344	−0.509	0.839	0.48	0.0003	0.373
*Experiment 3b: Spaced compound word—Identification of the Modifier lexeme *^*a*^							
L	1.184	0.624	−0.039	2.407	1.90	0.007	0.409
CFreq	0.393	0.626	−0.834	1.620	0.63	0.007	0.409
MFreq	**2.282**	**0.599**	**1.108**	**3.456**	3.81*	**0.007**	**0.409**
HFreq	−0.877	0.587	−2.028	0.274	−1.50	0.007	0.409
*Experiment 3b: Spaced compound word—Identification of the Head lexeme *^*a*^							
L	0.322	0.649	−0.95	1.594	0.50	0.003	0.381
CFreq	−0.397	0.650	−1.671	0.877	−0.61	0.003	0.381
MFreq	−0.431	0.623	−1.652	0.790	−0.69	0.003	0.381
HFreq	**1.590**	**0.610**	**0.394**	**2.786**	2.61*	**0.003**	**0.381**

orthographic length (L) and frequency (CFreq) of the compound word, word frequency of the modifier (MFreq) and head (HFreq). *SE* = Standard error. *Significant at the *p* = .05 level after Holm–Bonferroni method and bolded. ^a^ This model converged only with a random subject and item intercept and no predictor was included as part of a random slope.

**Table 3:** The baseline model + predictors for stimulus duration of correct responses in the progressive demasking task

Effect		Stimulus duration					
	*Β*	*SE*	2.5%	97.5%	*T*	*R*^2^*m*	*R*^2^*c*
*Experiment 1: Unspaced compound word PDT*^*a*^							
CFam	**−3.786**	**1.128**	**−5.997**	**−1.575**	−3.36*	**0.079**	**0.413**
CAoA	**7.725**	**1.107**	**5.555**	**9.895**	6.98*	**0.102**	**0.413**
CI	**7.969**	**1.026**	**5.958**	**9.98**	−7.77*	**0.108**	**0.412**
ST	−2.587	1.178	−4.896	−0.278	−2.20	0.073	0.413
LMD	−1.332	1.141	−3.568	0.904	−1.17	0.070	0.413
NA (H)	**4.099**	**1.154**	**1.837**	**6.361**	3.55*	**0.080**	**0.413**
NA (%)	**−4.413**	**1.151**	**−6.669**	**−2.157**	−3.83*	**0.081**	**0.413**
IA	−1.494	1.155	−3.758	0.77	−1.29	0.071	0.413
VC	1.689	1.136	−0.538	3.916	1.49	0.071	0.413
MFam	1.142	1.544	−1.884	4.168	0.74	0.070	0.413
MAoA	**4.545**	**1.367**	**1.866**	**7.224**	3.33*	**0.078**	**0.413**
MI	−2.804	1.133	−5.025	−0.583	−2.47	0.074	0.413
Hfam	−1.404	1.470	−4.285	1.477	−0.96	0.070	0.413
HAoA	2.307	1.352	−0.343	4.957	1.71	0.072	0.413
HI	−2.675	1.171	−4.970	−0.380	−2.28	0.073	0.413
*Experiment 2: Spaced compound word PDT*^*b,c*^							
CFam	**−2.447**	**0.625**	**−3.672**	**−1.222**	−3.91*	**0.008**	**0.507**
CAoA	**5.700**	**0.540**	**4.642**	**6.758**	10.55*	**0.026**	**0.507**
CI	**−5.129**	**0.538**	**−6.183**	**−4.075**	−9.54*	**0.024**	**0.507**
ST	−1.187	0.668	−2.496	0.122	−1.77	0.004	0.507
LMD	−1.343	0.635	−2.588	−0.098	−2.12	0.005	0.507
NA (H)	**2.146**	**0.653**	**0.866**	**3.426**	3.29*	**0.007**	**0.507**
NA (%)	**−2.652**	**0.642**	**−3.91**	**−1.394**	−4.13*	**0.009**	**0.507**
IA	−1.580	0.640	−2.834	−0.326	−2.47	0.005	0.507
VC	1.572	0.630	0.337	2.807	2.49	0.005	0.507
MFam	0.207	0.869	−1.496	1.91	0.24	0.003	0.507
MAoA	**2.702**	**0.765**	**1.203**	**4.201**	3.54*	**0.007**	**0.507**
MI	**−1.790**	**0.634**	**−3.033**	**−0.547**	−2.83*	**0.006**	**0.507**
Hfam	0.698	0.827	−0.923	2.319	−0.84	0.004	0.507
HAoA	1.903	0.751	0.431	3.375	2.54	0.005	0.507
HI	−1.354	0.660	−2.648	−0.06	−2.05	0.005	0.507
*Experiment 3a: Unspaced compound word- Identification of the Modifier lexeme *^*a*^							
CFam	**−0.869**	**0.338**	**−1.531**	**−0.207**	−2.58*	**0.001**	**0.410**
CAoA	0.378	0.369	−0.345	1.101	1.03	0.001	0.409
CI	0.127	0.351	−0.561	0.815	0.36	0.001	0.409
ST	0.202	0.344	−0.472	0.876	0.59	0.001	0.409
LMD	−0.211	0.325	−0.848	0.426	−0.65	0.001	0.409
NA (H)	−0.484	0.345	−1.16	0.192	1.40	0.001	0.409
NA (%)	−0.653	0.344	−1.327	0.021	−1.90	0.001	0.409
IA	−0.466	0.333	−1.119	0.187	−1.40	0.001	0.409
VC	−0.074	0.328	−0.717	0.569	−0.23	0.001	0.409
MFam	0.009	0.440	−0.853	0.871	0.02	0.001	0.409
MAoA	−0.297	0.405	−1.091	0.497	−0.73	0.001	0.409
MI	−0.020	0.329	−0.665	0.625	−0.06	0.001	0.409
Hfam	0.624	0.417	−0.193	1.441	1.49	0.001	0.409
HAoA	−0.052	0.388	−0.812	0.708	−0.14	0.001	0.409
HI	−0.432	0.339	−1.096	0.232	−1.28	0.001	0.409
*Experiment 3a: Unspaced compound word- Identification of the Head lexeme *^*a*^							
CFam	0.371	0.344	−0.303	1.045	1.08	0.0003	0.373
CAoA	−0.378	0.377	−1.117	0.361	−1.00	0.0003	0.373
CI	0.506	0.360	−0.200	1.212	1.40	0.0004	0.374
ST	0.504	0.354	−0.19	1.198	1.42	0.0005	0.373
LMD	−0.044	0.343	−0.716	0.628	−0.13	0.0003	0.373
NA (H)	0.079	0.355	−0.617	0.775	0.22	0.0003	0.373
NA (%)	−0.325	0.361	−1.033	0.383	−0.90	0.0004	0.373
IA	−0.110	0.345	−0.786	0.566	−0.32	0.0003	0.373
VC	−0.560	0.337	−1.221	0.101	−1.66	0.0010	0.373
MFam	0.315	0.459	−0.585	1.215	0.69	0.0003	0.373
MAoA	−0.322	0.419	−1.143	0.499	−0.77	0.0003	0.373
MI	0.538	0.346	−0.140	1.216	1.56	0.0010	0.373
Hfam	0.146	0.439	−0.714	1.006	0.33	0.0003	0.373
HAoA	−0.208	0.408	−1.008	0.592	−0.51	0.0003	0.373
HI	0.171	0.356	−0.527	0.869	0.48	0.0003	0.373
*Experiment 3b: Spaced compound word –Identification of the Modifier lexeme *^*a*^							
CFam	−1.310	0.580	−2.447	−0.173	−2.26	0.008	0.409
CAoA	**3.329**	**0.584**	**2.184**	**4.474**	5.70*	**0.015**	**0.409**
CI	**−3.559**	**0.540**	**−4.617**	**−2.501**	−6.59*	**0.017**	**0.409**
ST	−1.069	0.599	−2.243	0.105	−1.78	0.008	0.409
LMD	−0.897	0.574	−2.022	0.228	−1.56	0.007	0.409
NA (H)	**2.025**	**0.584**	**0.880**	**3.170**	3.47*	**0.010**	**0.409**
NA (%)	**−2.516**	**0.573**	**−3.639**	**−1.393**	−4.39*	**0.012**	**0.409**
IA	−1.289	0.578	−2.422	−0.156	−2.23	0.008	0.409
VC	0.469	0.576	−0.66	1.598	0.82	0.007	0.409
MFam	1.422	0.770	−0.087	2.931	1..85	0.008	0.409
MAoA	0.343	0.714	−1.056	1.742	0.48	0.007	0.409
MI	−0.006	0.583	−1.149	1.137	−0.01	0.007	0.409
Hfam	−0.339	0.742	−1.793	1.115	−0.46	0.007	0.409
HAoA	1.468	0.678	0.139	2.797	2.17	0.008	0.409
HI	**−1.828**	**0.581**	**−2.967**	**−0.689**	−3.15*	**0.010**	**0.409**
*Experiment 3b: Spaced compound word –Identification of the Head lexeme *^*a*^							
CFam	−0.986	0.608	−2.178	0.206	−1.62	0.004	0.381
CAoA	**3.909**	**0.587**	**2.758**	**5.060**	6.66*	**0.015**	**0.381**
CI	**−3.970**	**0.548**	**−5.044**	**−2.896**	−7.25*	**0.017**	**0.381**
ST	**−2.007**	**0.607**	**−3.197**	**−0.817**	−3.31*	**0.007**	**0.381**
LMD	−1.162	0.594	−2.326	0.002	−1.96	0.004	0.381
NA (H)	**1.930**	**0.611**	**0.732**	**3.128**	3.16*	**0.006**	**0.381**
NA (%)	**−1.804**	**0.616**	**−3.011**	**−0.597**	−2.93*	**0.006**	**0.381**
IA	−1.216	0.602	−2.396	−0.036	−2.02	0.004	0.381
VC	0.797	0.596	−0.371	1.965	1.34	0.004	0.381
MFam	0.869	0.807	−0.713	2.451	1.08	0.003	0.381
MAoA	1.907	0.725	0.486	3.328	2.63	0.005	0.381
** MI**	**−1.561**	**0.592**	**−2.721**	**−0.401**	**−2.64**	**0.005**	**0.381**
Hfam	−0.358	0.771	−1.869	1.153	−0.46	0.003	0.381
HAoA	0.568	0.715	−0.833	1.969	0.80	0.003	0.381
HI	−0.630	0.622	−1.849	0.589	−1.01	0.003	0.381

Conceptual familiarity of the compound word (CFam), age of acquisition of the compound word (CAoA), imageability of the compound word (CI), semantic transparency (ST), Lexeme meaning dominance (LMD), name agreement: H index (NA(H)) and percent (%), image agreement (IA) visual complexity (VC) of the compound word, familiarity of the modifier lexeme (MFam), Age of acquisition of the modifier lexeme (MAoA) and imageability of the modifier lexeme (MI). Familiarity of the head lexeme (Hfam), age of acquisition of the head lexeme (HAoA) and imageability of the head lexeme (HI). SE = Standard error. *Significant at the *p* = .05 level after Holm–Bonferroni method and bolded. ^a^ This model converged only with a random subject and item intercept and no predictor was included as part of a random slope ^b^ This model only converged for stimulus duration of correct responses when a random subject-intercept model is included ^c^ This model only converged for accuracy when a random subject-intercept model is included. Each predictor of interest was added independently to the baseline model.

However, there was no significant effect of length, compound word frequency, modifier frequency, semantic transparency, lexeme meaning dominance, or the familiarity, and imageability of the modifier and head lexemes. Likewise, the AoA of the head lexeme had no significant effect on unspaced PDT stimulus duration.

To evaluate the relative contributions of AoA and imageability to the identification threshold of unspaced compounds, we conducted a series of nested-model comparisons using likelihood ratio test and information criteria. We examined whether each variable provided a unique contribution to the model when the other was already present. Adding Imageability to a model already containing AoA resulted in a significant improvement, χ^2^(4) = 37.29, *p* < .001, reducing the AIC by 30 units (from 67,455 to 67,425). Similarly, adding AoA to a model containing imageability also provided a significant improvement, χ^2^(4) = 35.42, *p* < .001, reducing the AIC by 28 units (from 67,453 to 67,425). The results for unspaced compound revealed a more balanced contribution from both variables and provide roughly equivalent and independent predictive power. 

## Experiment 2: Spaced progressive demasking

Consistent with prior research on compound word processing (Elsherif & Catling, 2023, 2024; Juhasz et al., 2015), our Experiment 1 revealed that participants were faster at identifying unspaced compound words that were acquired earlier, were more familiar and were more imageable. Notably, we also found that the AoA of the modifier and the compound word's name agreement both influenced identification speed.

Experiment 2 explored whether AoA effects also influenced the processing of spaced compound words. Presenting compound words with a space is a subtle methodological change, yet prior research suggests it encourages morphological decomposition, forcing readers to process the words morphemically (e.g., Brooks & Cid de Garcia, 2015; Elsherif et al., 2020; Frisson et al., 2008). This decomposition is believed to require more effort to integrate the meaning of the individual morphemes, potentially leading to enhanced semantic processing. Consequently, factors like AoA, imageability, familiarity, and frequency are expected to have a stronger impact on the speed of compound word recognition (e.g., Elsherif et al., 2020; Elsherif & Catling, 2023). Therefore, we also compared the progressive demasking for unspaced versus spaced words with test the hypothesis that the morphological decomposition route promotes additional semantic processing during visual word recognition. This is an extension of prior work (e.g., Elsherif & Catling, 2023) and we hypothesized that inserting a space between morphemes would trigger morphological decomposition, leading to increased semantic processing to combine the morphemes. This, in turn, should result in observable effects of morphemic AoA, as well as a stronger influence of both AoA and other semantic factors.

### Method

#### Participants

Participants were 48 British undergraduates at the University of Leicester, who earned course credit for their participation. The students were between 18 and 21 years of age (*M*_age_ = 18.81 ± 0.69 years; 26 women). All participants were monolingual, had normal or corrected-to-normal vision and had signed a consent form to participate in the study. None had participated in Experiment 1. All participants were native English speakers.

#### Stimuli, procedure and analyses

The experimental word stimuli, experimental procedures and analyses were the same as in Experiment 1, except that the participants saw spaced compound words (e.g., ‘air plane’) instead of unspaced compound words (e.g., ‘airplane’).

#### Spaced PDT

Following the advice of Woods et al. (2023), the rate of missingness across the samples was less than 1% and was considered missing at random. The average stimulus duration was 71.4 ms (*SD* = 37.4, 95% CI [70.6, 72.2]). The average stimulus duration was also significantly shorter for spaced compound words than for unspaced compound words (*b* = 7.77, *SE* = 3.67, *t* = 2.12, *p* = .04).

We investigated the impact of word frequency, AoA, familiarity, semantic transparency, imageability, LMD, and morphemic properties on stimulus duration for the spaced PDT. In the baseline model, we observed a significant effect of the compound word frequency on stimulus duration rates. High-frequency spaced compound words took less time to identify than low-frequency ones (Table 2).

Additionally, we found that several other factors influenced stimulus duration. The AoA of a compound word was a significant predictor, as spaced compound words acquired earlier in life were identified more quickly. Name agreement (both H index and percentage) also significantly influenced stimulus duration, with compounds that had higher agreement and less spread being identified more rapidly (Table 3). Furthermore, both familiarity and imageability of the compound word were significant predictors; higher familiarity and imageability were associated with faster identification times. We also found that the AoA of the modifier lexemes influenced stimulus duration, with earlier-acquired modifiers leading to faster identification of the whole compound. The imageability of the modifier was a significant contributor, with a more imageable modifier being associated with faster identification of the spaced compound word.

However, several variables had no significant effect on unspaced PDT stimulus duration. These included length, compound-word frequency, modifier frequency, semantic transparency, lexeme-meaning dominance and the familiarity of the modifier and head lexemes, as well as the imageability and AoA of the head lexeme.

To evaluate the relative contributions of AoA and imageability to the identification threshold of spaced compounds, we conducted a series of nested-model comparisons using likelihood ratio test and information criteria. We examined whether each variable provided a unique contribution to the model when the other was already present. When AoA was added to a model already containing imageability, it resulted in a massive and highly significant improvement in model fit, χ^2^(4) = 153.77, *p* < .001, reducing the AIC by 146 units (from 65,758 to 65,612). Conversely, when imageability was added to a model already containing AoA, the improvement was significantly smaller, though still statistically significant, χ^2^(4) = 25.54, *p* < .001, reducing the AIC by only 17 units (from 65629 to 65,612). These results demonstrate that while both variables explain unique variance, AoA is a substantially more robust predictor of duration thresholds for spaced compounds. The six-fold difference in χ^2^ improvement and the vastly larger reduction in AIC confirm the AoA is a much more robust predictor of duration thresholds for spaced compounds than imageability.

**Fig. 2:**
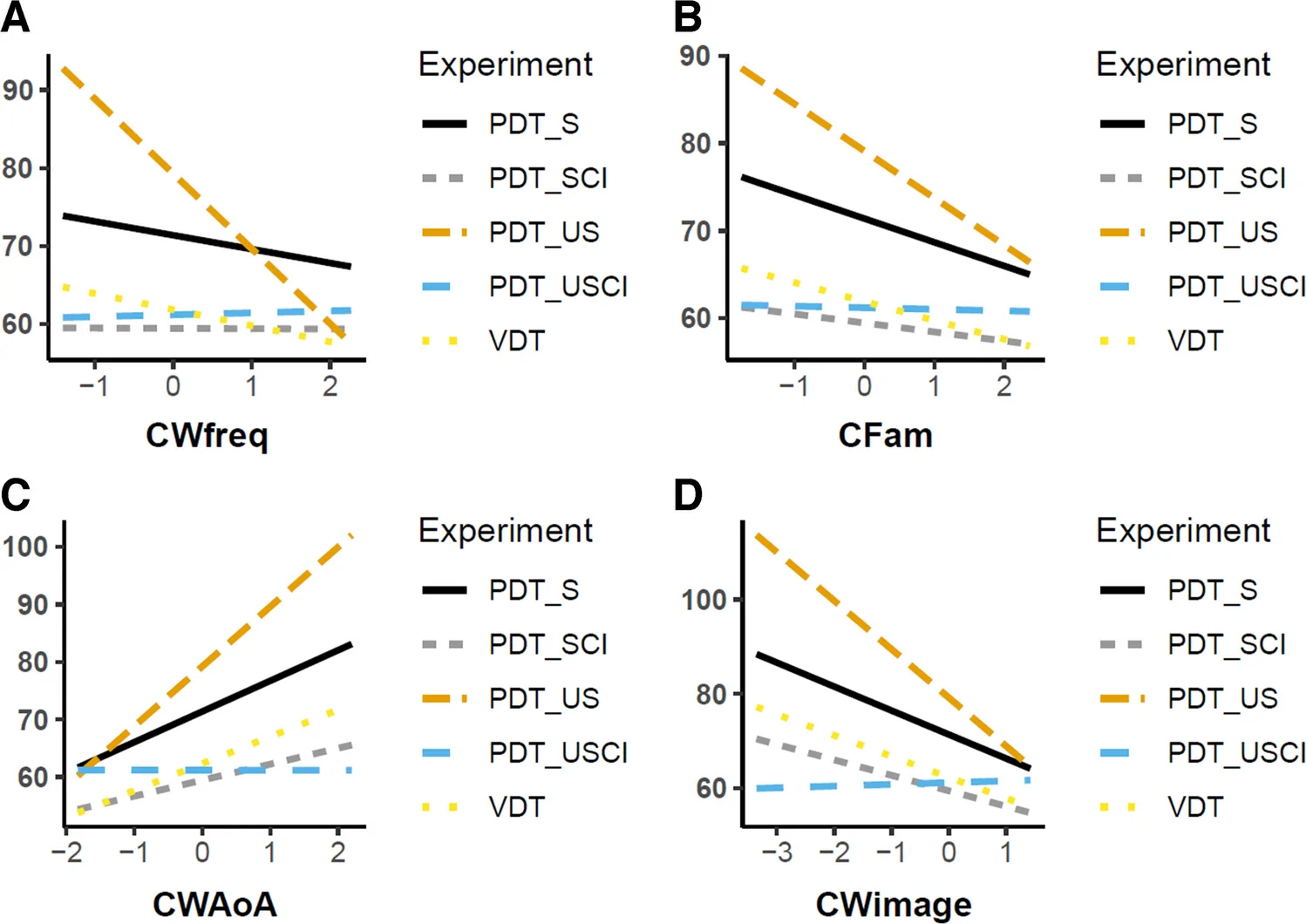
The interaction between (**A**) frequency, (**B**) familiarity, (**C**) AoA and (**D**) imageability and the different tasks on stimulus duration. *Note.* CWfreq is frequency of the compound word; CFam is familiarity of the compound word; CWAoA is the AoA of the compound word; CWimage is the imageability of the compound word; PDT_S is spaced compound words in progressive demasking; PDT_US is unspaced compound words in progressive demasking task; PDT_SCI is progressive demasking task spaced compound words for component identification; PDT_USCI is progressive demasking task unspaced compound words for component identification; VDT is visual duration threshold. (Colour figure online)

As Juhasz et al. (2015) noted, the familiarity and imageability of a compound word are key indicators of lexical-semantic processes. We observed these factors to be greater for unspaced compound words (familiarity of unspaced compound word: *b* = −3.79; imageability of the compound word: 7.97), compared with spaced compound words (familiarity of spaced compound word: *b* = −.45, imageability of the compound word: *b* = −5.13; see Table 3 and Fig. 2). This finding suggests that the process of accessing and using the meaning of a compound word may be more robust when the words appear as a single unit rather than as separate constituents. Furthermore, while the AoA of the individual morphemes (e.g., the modifier and head), together with the imageability of the modifier all affected stimulus duration threshold for spaced compound words but not unspaced compound words. This suggests that the processing of the component parts is more pronounced when they are visually separated. In contrast to this morphemic-level difference, we observed a consistent whole-word AoA effect across both spaced and unspaced tasks in Experiments 1 and 2: participants identified early-acquired compound words more quickly than late-acquired ones. This finding replicates previous studies on AoA effects in visual word identification (e.g., Catling et al., 2008; Chen et al., 2009; Dent et al., 2007), confirming that the AoA of a compound word as a single unit is a robust predictor of visual word identification.

Our findings, while not showing a larger AoA effect for spaced than unspaced compound words (see Table 3), still support the integrated account of lexical access (e.g., see Elsherif et al., 2023, for a review). Progressive demasking involves the initial identification of a word string, which activates its lexical representation through information about word form and spelling. This lexical access then triggers the feed-forward activation of the semantic representation (orthography-to-semantics; Ploetz & Yates, 2016). This study provides further evidence for this notion, as we found shorter stimulus durations for words that were more familiar, highly frequent and more imageable. These characteristics are likely to trigger additional semantic processing, which facilitates word identification. The integrated account explains this as a result of competition between the morphemic constituent and the compound word, as well as the less systematic mappings between letters and sounds (Y.-N. Chang et al., 2019). Thus, we can conclude that, like monosyllabic items, the AoA effect of both the morpheme and the compound word contributes to compound word recognition.

## Experiments 3a and 3b: Spaced and unspaced progressive demasking with component Identification

The AoA effect for compound words has been a focus of research on spaced and unspaced compound word processing (Elsherif et al., 2020; Elsherif & Catling, 2023, 2024). Ideally, we should assess the individual components that form a compound word—more specifically, the modifier and head lexemes. The logic behind this is that if we have the reader extract the modifier or head, we force them to travel via the decomposition route, whilst inhibiting the direct lexical route. Brooks and Cid de Garcia (2015) examined the neural processing of different word types, specifically transparent compounds (e.g., ‘roadside’), opaque compounds (e.g., ‘butterfly’), and morphologically simple words (e.g., ‘spinach’). They used a word-naming task with a partial-repetition priming paradigm, with their analysis focused on the visual processing of compound word primes. The authors observed that, relative to morphologically simple words, transparent compound words elicited greater activation in the anterior middle temporal gyrus approximately 250–470 ms poststimulus, with stronger effects observed in the posterior superior temporal gyrus between 430 and 600 ms. Brooks and Cid de Garcia concluded that the processing of compound words involves a decomposition stage that is independent of semantic meaning. Elsherif and Catling (2021) asked participants to read either the modifier (e.g., ‘air’) or the head lexeme (e.g., ‘plane’) of a compound word (e.g., ‘airplane’) depending on a number presented above the word. For instance, if a '1' was presented, participants had to read ‘air’, while if a '2' was presented, they had to read ‘plane’. The authors observed that when participants were forced to read the modifier, effects were seen for the AoA, imageability, and familiarity of the compound word, along with the AoA of the modifier. However, when participants were forced to read the head lexeme, only effects of AoA, imageability, and familiarity of the compound word were observed. The authors argued that the modifier is processed prior to the head lexeme, which requires direct access to the lexical representations when processed. We investigate how the individual components of a compound word are identified with increasing signal-to-noise ratio (SNR). In this progressive demasking task, the ‘signal’ is the clarity of the individual morpheme or head, while the ‘noise’ is the visual mask. As the SNR increases, the target word gradually becomes clearer and easier to perceive over time, while the other component remains static. We therefore predict that an AoA effect of the compound word should be observed during the identification of both the modifier and head lexeme in both unspaced and spaced lexemes.

### Method

#### Participants

Participants were 96 British undergraduates at the University of Leicester split between Experiments 3a and 3b, who earned course credit for their participation. The students were between 18 and 21 years of age (Experiment 3a: *M*_age_ = 19.42 ± 1.54 years; 28 women; Experiment 3b: *M*_age_ = 18.27 ± 1.30 years; 34 women). All participants were monolingual, had normal or corrected-to-normal vision and had signed a consent form to participate in the study. None had participated in either Experiments 1 or 2. Participants in Experiment 3a did not participant in Experiment 3b or vice versa. All participants were native English speakers and had normal or corrected-to-normal vision.

#### Stimuli, procedure and analyses

The experimental word stimuli, procedures, and analyses for Experiment 3a were identical to those in Experiments 1 and 2, with the following exceptions: participants were instructed to identify either the compound word or one of its components. They saw unspaced compound words, but only one of its components alternated with the pattern mask. The component that alternated was either the modifier (e.g., ‘air’) or the head lexeme (e.g., ‘plane’); the other component remained visible throughout the trial. Experiment 3b followed the same procedures as Experiment 3a, except that participants saw spaced compound words instead of unspaced ones.

### Results

#### Experiment 3a: Unspaced progressive demasking with identification of the modifier component

Following the advice of Woods et al. (2023), the rate of missingness across the samples was less than 1% and was considered missing at random. The average stimulus duration for identifying the modifier was 59.1 ms (*SD* = 31.1, 95% CI [58.4, 59.8]). We found that PDT with modifier or head component identification for unspaced compound words was identified earlier than PDT for the whole compound word with spaced compound words (*b* = −10.04, *SE* = 3.66, t = −2.74, *p* = .007) and PDT for the whole compound word with unspaced compound words (*b* = −17.80, *SE* = 3.66,* t* = −4.86, *p* < .001).

We investigated the impact of word frequency, AoA, familiarity, semantic transparency, imageability, lexeme meaning dominance (LMD), and morphemic properties on stimulus duration for unspaced PDT. Here we observed only the familiarity of the compound word influenced stimulus duration, with unspaced compound words that had higher familiarity being identified faster.

However, we found no significant effect of length, compound word frequency, modifier or head frequency, name agreement, image agreement, AoA and imageability of the compound word. Similarly, the AoA, imageability, and familiarity of the modifier and head lexemes had no significant effect on the stimulus duration for identifying the modifier component in unspaced PDT.

To evaluate the relative contributions of AoA and imageability to the identification threshold of unspaced compounds for the modifier lexeme, we further examined the relative contributions of AoA and Imageability for the modifier lexemes within the unspaced PDT condition using nested-model comparisons. When imageability was added to a model already containing AoA, it resulted in a nonsignificant improvement in model fit, χ^2^(1) = 3.14, *p* = .08, reducing the AIC by one unit (from 66,710 to 66,709). When AoA was added to the model already containing imageability, it resulted in a significant improvement in model fit, χ^2^(1) = 4.07, *p* = .04), reducing the AIC by two units. Although the effect sizes for constituents in the unspaced condition are smaller than those observed for the unspaced and spaced compound, these results further support the primacy of acquisition age.

#### Experiment 3a: Unspaced progressive demasking with identification of the head component

Following the advice of Woods et al. (2023), the rate of missingness across the samples was less than 1% and considered missing at random. The average stimulus duration for identifying the head was 59.8 ms (*SD* = 31.4, 95% CI [59.1, 60.5]). There was no significant difference between the modifier and the head for unspaced compound words (*b* = 0.077, *SE* = 0.46, *t* = 1.66, *p* = .10). We investigated the impact of word frequency, AoA, familiarity, semantic transparency, imageability, LMD, and morphemic properties on stimulus duration for unspaced PDT. In both identification latencies of the head lexeme, we found no significant influence from frequency, familiarity, AoA, or the imageability of the compound word, head, or modifier. Similarly, orthographic length and the semantic transparency of the compound word did not have a significant effect on stimulus duration (see Tables 2 and 3).

To evaluate the relative contributions of AoA and imageability to the identification threshold of unspaced compounds for the head lexeme, we further examined the relative contributions of AoA and Imageability for the head lexemes within the unspaced PDT condition using nested-model comparisons. Adding imageability to the AoA model did not significantly improve fit, χ^2^(1) = 0.98, *p* = .32, and similarly, adding AoA to the Imageability model produced no significant improvement, χ^2^(1) = 0.01, *p* = .91. The AIC values for all models remained within 1–2 units of each other (approx. 66,905).

#### Experiment 3b: Spaced progressive demasking with identification of the modifier component

Following the advice of Woods et al. (2023), the rate of missingness across samples was less than 1% and considered missing at random. The average stimulus duration for identifying the modifier was 60.81 ms (*SD* = 34.85, 95% CI [59.99, 61.62]). We found that PDT with modifier or head component identification averaged together was significantly faster than progressive demasking of the whole compound word for both spaced (*b* = 11.98, *SE* = 3.66, *t* = 3.27, *p* = .0012) and unspaced compound words (*b* = 19.75, *SE* = 3.66, *t* = 5.39, *p* < .001). However, there was no significant difference between component identification for spaced and unspaced compound words (*b* = 1.95, *SE* = 3.66, *t* = 0.53, *p* = .59).

In the baseline model, we observed a significant effect of modifier frequency on stimulus duration, such that spaced compound words with higher-frequency modifiers were identified faster than those with low-frequency modifier lexemes (Table 2). Additionally, name agreement (both H index and percentage) significantly influenced the stimulus duration for identifying the modifier component (Table 3). Spaced compound words with higher name agreement and less spread were identified more quickly. The AoA and imageability of the compound word also influenced the stimulus duration for identifying the modifier component (Table 3). Specifically, compound words acquired earlier in life and those that were more imageable took less time to identify the modifier in spaced compound words. In addition, the imageability of the head lexeme influenced the stimulus duration for identifying the modifier component (Table 3), with spaced compound words having a more imageable head lexeme being identified more quickly. However, no significant effects were found for length, compound word frequency, modifier frequency, image agreement, familiarity of the compound word, semantic transparency, lexeme meaning dominance, or the AoA and familiarity of the modifier and head lexemes, together with the imageability of the modifier lexeme, on the stimulus duration for identifying the modifier component during spaced PDT.

To evaluate the relative contributions of AoA and imageability to the identification threshold of spaced compounds for the modifier lexeme, we further examined the relative contributions of AoA and imageability for the modifier lexemes within the spaced PDT condition using nested-model comparisons. When imageability was added to a model already containing AoA, it resulted in a massive and highly significant improvement in model fit, χ^2^(1) = 21.45, *p* < .001, reducing the AIC by 20 units (from 65,725 to 65,705). Conversely, when AoA was added to a model already containing imageability, the improvement was significantly smaller, though still statistically significant, χ^2^(1) = 10.64, *p* < .001, reducing the AIC by only nine units (from 65,714 to 65,705). These results demonstrate that while both variables explain unique variance, imageability is a substantially more robust predictor of duration thresholds for identifying the modifier lexeme in spaced compounds. The twofold difference in χ^2^ improvement and the vastly larger reduction in AIC confirm the imageability is a much more robust predictor of duration thresholds for spaced compounds than AoA.

### Experiment 3b: Spaced progressive demasking with identification of the head component

Following the advice of Woods et al. (2023), the rate of missingness across samples was less than 1% and considered missing at random. The average stimulus duration for identifying the head was 61.60 ms (*SD* = 34.97, 95% CI [60.78, 62.42]). No significant difference was found between the components for spaced compound words (*b* = 0.061, *SE* = 0.42, *t* = 1.48, *p* = .14).

We investigated the impact of word frequency, AoA, familiarity, semantic transparency, imageability, LMD, and morphemic properties on stimulus duration for spaced component identification. In the baseline model, we observed a significant effect of the head's frequency, with unspaced compound words that had higher head frequency requiring less time to identify the head lexeme (Table 2).

Furthermore, name agreement (both H index and percentage) significantly influenced stimulus duration during head component identification (Table 3), with the identification of the head lexeme in spaced compound words that had higher agreement and less spread being identified more quickly. The AoA and imageability of the compound word also influenced the stimulus duration of the head component, as identification of the head lexeme in spaced compound words was quicker with compound words that were acquired earlier and those that were more imageable. Semantic transparency influenced head component identification, with more transparent spaced compound words being identified faster. However, we found no significant effect of length; compound-word frequency; modifier frequency; image agreement; familiarity of the compound, modifier, or head lexemes; lexeme-meaning dominance or the AoA and imageability of the modifier and head lexemes on the identification of the head component during spaced component identification.

To evaluate the relative contributions of AoA and imageability to the identification threshold of spaced compounds for the head lexeme, we further examined the relative contributions of AoA and Imageability for the head lexemes within the spaced PDT condition using nested-model comparisons. When AoA was added to a model already containing imageability, it resulted in a massive and highly significant improvement in model fit, χ^2^(4) = 25.68, *p* < .001, reducing the AIC by 18 units (from 66,007 to 65,989). Conversely, when imageability was added to a model already containing AoA, the improvement was significantly smaller, though still statistically significant, χ^2^(4) = 12.12, *p* = .02, reducing the AIC by only four units (from 65,993 to 65,989). These results demonstrate that while both variables explain unique variance, AoA is a substantially more robust predictor of duration thresholds for identifying the head lexeme in spaced compounds. The fourfold difference in χ^2^ improvement and the vastly larger reduction in AIC confirm the AoA is a much more robust predictor of duration thresholds for spaced compounds than imageability.

In Experiment 3a, we found no effects of psycholinguistic predictors for unspaced compound words. However, an AoA effect was observed in the spaced compound words for component word identification in Experiment 3b: participants took less time to identify early-acquired compared with late-acquired words. The findings from these tasks are novel: although the results for spaced compound words replicate those of Elsherif and Catling (2021), the findings for unspaced compound words do not replicate previous research. One possible explanation is that, unlike unspaced compounds, spaced compound words require additional processing of relational semantics to combine their constituent lexemes. Unspaced compounds are more regular and systematic in nature. The mapping between lexical and lexemic representations becomes more consistent and regular, allowing late-acquired words to exploit the structure formed by early-acquired words (see Ellis & Lambon Ralph, 2000). As a result, focusing on component identification makes it easier to separate the components into their respective head and modifier lexemes, which means late-acquired words do not induce an additional processing cost. These findings provide evidence in favour of the mapping theory subsumed under the integrated account (Elsherif et al., 2023). We can thus argue that the AoA effect influences spaced compound words, like the findings of Elsherif and Catling (2021), but not unspaced compound words.

## Experiment 4: Visual Duration Threshold

Finally, we need to assess whether the AoA and constituent effects are localised strictly in the morpho-orthographic system or at a broader perceptual-semantic mapping by comparing responses with word and pictorial stimuli. By using objects that share the same conceptual identity as the compound words in Experiments 1–3, we can investigate whether the modifier and head lexemes continue to influence the identification threshold even when orthographic structure is removed. While the AoA effect may stem from the arbitrary mapping between different levels of representation in compound words, it is consistently shown to be much larger in tasks with more irregular and unpredictable mapping, such as object recognition and naming (e.g., Lambon-Ralph & Ehsan, 2006). According to the mapping theory (Y.-N. Chang et al., 2019), AoA effects are most pronounced in tasks with irregular and unpredictable mapping such as in tasks with irregular and unpredictable mapping, such as object recognition and naming.

Additionally, the AoA effect is less likely to be observed during word naming than picture naming (Belke et al., 2005). Belke et al. (2005) found that when AoA was entered into a stepwise regression model, it accounted for 56.4% of the variance in response times for picture naming, whereas word frequency only added 0.6%. When word frequency was entered first, it accounted for 22.8% of the variance, but this increased significantly to 57% when AoA was subsequently added. In contrast, for word naming, AoA explained only 0.7% of the variance, and word frequency increased this to just 17%. This suggests that the AoA effect is less pronounced for items with systematic and regular mapping but is consistently demonstrated for pictorial items (Perret & Bonin, 2019). As a result, we specifically sought to compare compound words to objects to assess if the magnitude of the AoA effect is similar. If this prediction is true, it suggests that compound words, like objects, possess a degree of irregular mapping that makes them more susceptible to the AoA effects. However, there has been limited research on the AoA effect especially in compound words in visual duration thresholds. If the constituent AoA remains a significant predictor in VDT, it demonstrates that these effects are not merely linguistic artefacts but are situated at a perceptual-semantic locus where organised sets of visual features map onto known morphological types. Given that compound words are often more irregular and less systematic in their mapping, the AoA effects are likely to be comparable to those seen in picture naming. This would allow us to investigate the nuances of how the AoA effect arises.

### Method

#### Participants

Participants were 48 British undergraduates at the University of Leicester for Experiment 4, who earned course credit for their participation. The students were between 18 and 21 years of age (*M*_age_ = 18.81 ± 0.81 years; 26 women). All participants were monolingual, had normal or corrected-to-normal vision and had signed a consent form to participate in the study. None had participated in the previous experiments. All participants were native English speakers.

#### Stimuli, procedures and analyses

Experiment 4 used the same procedures, and analyses as Experiments 1–3b, but instead of words, we presented participants with pictures of the same 150 compound words and used a VDT as opposed to a PDT. The goal was to determine the minimum time needed for participants to correctly identify a masked pictorial stimulus. All pictures were sized at 300 × 300 pixels and were immediately preceded by a visual pattern mask of the same size. This mask, made from a random dot pattern, was designed to prevent participants from seeing the image for too long. Each dot (1 pixel) was about 0.294 mm high and 0.294 mm wide. The images and the mask were black and white.

Consistent with prior research on standardised stimulus sets, participants were familiarized with the experimental pictures before beginning the tasks (e.g., Damian & Dumay, 2007). During this phase, each picture was shown with its designated name to ensure participants recognized the visual stimuli and to promote consistent naming of compound words (e.g., ‘baby carriage’) rather than monomorphemic alternatives (e.g., ‘pram’) in the subsequent visual duration threshold task.

To ensure a stable refresh rate of 60Hz, the study began with an FPS counter. The trial procedure began with a 2,000-ms fixation cross, followed by a 2,000-ms visual mask. The target image was then presented for a varying amount of time, after which a second mask was displayed for 2,000-ms. Participants were then cued to identify the object after the second mask. They were encouraged to guess if they were unsure.

On the first display the target item was presented for 50.00 ms. Following the second mask, participants were given 2,000 ms to state the name of the object. If participants correctly identified the object, they moved to the next trial with the experimenter clicking a tick button with the mouse to record a correct response. If not, the experimenter clicked a cross button and the participant was allowed to see the image again for a longer duration. The image duration was increased by increments of 16.67 ms, up to a maximum of 116.67 ms. Participants also had the option to indicate that they could identify the object but did not know the name of the object; in this case, the experimenter clicked a question mark button to indicate a lack of recognition. The order in which objects were presented was randomized to minimize practice effects. The visual duration threshold was defined as the shortest exposure time at which a participant could correctly identify an object.

### Results

#### Accuracy

Following the advice of Woods et al. (2023), the rate of missingness across the samples was less than 1% and was considered missing at random. The average accuracy was 0.88 (*SD* = 0.32, 95% CI [0.873, 0.889]). We investigated the impact of word frequency, AoA, familiarity, semantic transparency, imageability, lexeme meaning dominance (LMD), and morphemic properties on accuracy for VDT. In the baseline model, we observed a significant effect of head and compound word frequency on accuracy, such that higher-frequency head lexemes and compound words were identified more accurately (Table 4).

**Table 4:** The baseline model results for accuracy and stimulus duration of correct responses in the visual duration threshold task

Effect		Accuracy							Stimulus duration					
	*β*	*SE*	2.5%	97.5%	*T*	*R*^2^*m*	*R*^2^*c*	*β*	*SE*	2.5%	97.5%	*T*	*R*^2^*m*	*R*^2^*c*
*Visual Duration Threshold*^*a*^														
L	0.747	1.906	−2.989	4.483	0.39	0.080	0.590	0.375	0.846	−1.283	2.033	0.44	0.027	0.421
CFreq	**0.593**	**0.194**	**0.213**	**0.973**	**3.06**	**0.080**	**0.590**	−1.674	0.850	−3.34	−0.008	−1.97	0.027	0.421
MFreq	0.050	0.179	−0.301	0.401	0.28	0.080	0.590	−0.258	0.814	−1.853	1.337	0.32	0.027	0.421
HFreq	**0.452**	**0.182**	**0.095**	**0.809**	**2.48**	**0.080**	**0.590**	**−1.903**	**0.793**	**−3.457**	**−0.349**	−2.40*	**0.027**	**0.421**

orthographic length (L) and frequency (CFreq) of the compound word, word frequency of the modifier lexeme (MFreq) and word frequency of the head lexeme (HFreq). *SE* = standard error. *Significant at the *p* = .05 level after Holm–Bonferroni method and bolded. ^a^ This model converged only with a random subject and item intercept and no predictor was included as part of a random slope ^b^ This model only converged for stimulus duration of correct responses when a random subject-intercept model is included ^c^ This model only converged for accuracy when a random subject-intercept model is included.

Additionally, name agreement (both H index and percentage) and image agreement significantly influenced the accuracy of object identification (Table 5). Objects with higher image and name agreement, as well as less spread in name agreement, were recognized more accurately. The familiarity of the compound word also influenced the accuracy of VDT, with more familiar objects being recognized more accurately. The AoA and imageability of the compound word were significant factors in VDT accuracy (Table 5). Objects acquired earlier in life and those that were more imageable were identified more accurately. Similarly, the AoA and imageability of the modifier influenced VDT accuracy, with objects having earlier-acquired and more imageable modifiers being recognized more correctly. The imageability of the head lexeme also influenced object identification accuracy; objects with a more imageable head being recognized more correctly than those with a less imageable one.

**Table 5:** The baseline + predictors model results for accuracy and stimulus duration of correct responses in the visual duration threshold task

Effect			Accuracy								Stimulus duration						
	*Β*		*SE*	2.5%	97.5%	*T*	*R*^2^*m*	*R*^2^*c*		*β*	*SE*	2.5%	97.5%	*T*	*R*^2^*m*		*R*^2^*c*
*Visual Duration Threshold*^*a*^																	
CFam	**0.704**		**0.173**	**0.365**	**1.043**	4.06*	**0.134**	**0.587**		−2.004	0.782	−3.537	−0.471	−2.56	0.394		0.422
CAoA	**−1.445**		**0.158**	**−1.755**	**−1.135**	−9.16*	**0.278**	**0.575**		**5.345**	**0.758**	**3.859**	**6.831**	7.06*	**0.095**		**0.416**
CI	**1.163**		**0.145**	**0.879**	**1.447**	8.01*	**0.224**	**0.567**		**−4.351**	**0.763**	**−5.846**	**−2.856**	−5.70*	**0.069**		**0.415**
ST	0.427		0.182	0.070	0.784	2.34	0.098	0.586		−1.060	0.813	−2.653	0.533	−1.30	0.030		0.423
LMD	0.317		0.180	−0.036	0.670	1.76	0.093	0.590		−0.844	0.780	−2.373	0.685	−1.08	0.030		0.422
NA (H)	**−0.988**		**0.167**	**−1.315**	**−0.661**	−5.93*	**0.184**	**0.587**		**2.733**	**0.789**	**1.187**	**4.279**	3.46*	**0.048**		**0.420**
NA (%)	**0.956**		**0.168**	**0.627**	**1.285**	5.68*	**0.175**	**0.587**		**−2.898**	**0.788**	**−4.442**	**−1.354**	−3.68*	**0.050**		**0.420**
IA	**0.923**		**0.170**	**0.59**	**1.256**	5.43*	**0.177**	**0.596**		**−3.876**	**0.720**	**−5.287**	**−2.465**	−5.38*	**0.074**		**0.419**
VC	−0.453		0.170	−0.786	−0.120	−2.66	0.106	0.586		**2.595**	**0.756**	**1.113**	**4.077**	3.43*	**0.049**		**0.422**
MFam	−0.105		0.242	−0.579	0.369	−0.44	0.081	0.591		0.312	1.058	−1.762	2.386	0.29	0.043		0.431
MAoA	**−0.724**		**0.204**	**−1.124**	**−0.324**	−3.54*	**0.119**	**0.585**		**3.925**	**0.919**	**2.124**	**5.726**	4.27*	**0.056**		**0.420**
MI	**0.515**		**0.170**	**0.182**	**0.848**	3.03*	**0.111**	**0.592**		**−2.496**	**0.764**	**−3.993**	**−0.999**	−3.26*	**0.046**		**0.422**
Hfam	0.359		0.226	−0.084	0.802	1.59	0.089	0.590		−0.488	1.011	−2.47	1.494	−0.48	0.028		0.423
HAoA	−0.577		0.211	−0.991	−0.163	−2.74	0.108	0.592		2.112	0.913	0.323	3.901	2.31	0.037		0.421
HI	**0.485**		**0.174**	**0.144**	**0.826**	2.78*	**0.106**	**0.589**		**−2.304**	**0.793**	**−3.858**	**−0.75**	−2.91*	**0.042**		**0.421**

Conceptual familiarity of the compound word (CFam), age of acquisition of the compound word (CAoA), imageability of the compound word (CI), semantic transparency (ST), lexeme meaning dominance (LMD), name agreement: H index (NA(H)) and percent (%), image agreement (IA) visual complexity (VC) of the compound word, familiarity of the modifier lexeme (MFam), age of acquisition of the modifier lexeme (MAoA) and imageability of the modifier (MI). Familiarity of the head lexeme (Hfam), age of acquisition of the head lexeme (HAoA) and imageability of the head lexeme (HI). *SE* = standard error. *Significant at the *p* = .05 level after Holm–Bonferroni method and bolded. ^a^ This model converged only with a random subject and item intercept and no predictor was included as part of a random slope ^b^ This model only converged for stimulus duration of correct responses when a random subject-intercept model is included ^c^ This model only converged for accuracy when a random subject-intercept model is included.

However, several factors had no significant effect on the accuracy of identifying the modifier component in VDT. These included length, modifier and head frequency, semantic transparency, lexeme meaning dominance, and the familiarity of the modifier and head lexemes, as well as the AoA of the head lexeme.

#### Stimulus duration

Following the advice of Woods et al. (2023), the rate of missingness across the samples was less than 1% and was considered missing at random. The average stimulus duration was 61.77 ms (*SD* = 16.87, 95% CI [61.36, 62.19]). VDT stimuli were identified more quickly than PDT stimuli for unspaced compound words (*b* = 16.64, *SE* = 3.67, *t* = 4.53, *p* < .001) and spaced compound words (*b* = 8.88, *SE* = 3.67, *t* = 2.42, *p* = .02). However, there was no significant difference between VDT and PDT with component identification for unspaced compound words (*b* = −1.16, *SE* = 3.67, *t* = −0.32, *p* = .75), or between VDT and PDT with spaced compound words (*b* = −3.11, *SE* = 3.67,* t* = −0.85, *p* = .40).

We investigated the impact of word frequency, AoA, familiarity, semantic transparency, imageability, LMD and morphemic properties on stimulus duration for VDT. In the baseline model, we observed a significant effect of the head lexeme frequency on stimulus duration, such that objects with a higher head frequency were identified faster (Table 4). Additionally, both name agreement (H index and percentage) and image agreement significantly influenced the object’s stimulus duration (Table 5). Objects with higher image agreement, higher name agreement, and less spread in name agreement were identified more quickly. The visual complexity of the image also influenced stimulus duration, with objects that had higher visual complexity taking longer to identify. The AoA and imageability of the compound word influenced the stimulus duration of the object’s identification (Table 5). Objects acquired earlier in life and those that were more imageable were identified more quickly. Additionally, the AoA and imageability of the modifier influenced the stimulus duration of the object. Objects with modifiers acquired earlier in life and that were more imageable were identified more quickly. Furthermore, the imageability of the head lexeme influenced the stimulus duration of the object’s identification, with more imageable heads being associated with taking less time to identify.

However, there was no significant effect on the VDT stimulus duration of the following variables: length, modifier and compound word frequency, semantic transparency, lexeme meaning dominance or the familiarity of the modifier and head lexemes and the AoA of the head lexeme.

To evaluate the relative contributions of AoA and imageability to the identification threshold of pictures, we further examined the relative contributions of AoA and imageability for VDT using nested-model comparisons. When AoA was added to a model already containing imageability, it resulted in a massive and highly significant improvement in model fit, χ^2^(4) = 391.27, *p* < .001, reducing the AIC by 383 units (from 50,960 to 50,577). In contrast, adding imageability to a model containing AoA provided a comparatively negligible, though statistically significant, improvement, χ^2^(4) = 18.05, *p* = .001, with an AIC reduction of only 10 units (from 50,587 to 50,577). These findings provide definitive evidence that at the earliest stages of perceptual identification, the AoA is the primary driver of the identification threshold. The 383-unit reduction in AIC suggests that AoA captures a fundamental aspect of lexical accessibility that mageability cannot account for, effectively addressing concerns regarding the distinguishability of these two factors in our stimulus set. These results across tasks, using a likelihood ratio test for AIC and BIC, are summarized in Table 6.

**Table 6:** Summary of nested model comparisons for the independent effects of AoA and imageability

Task	Added Predictor	*df*	χ^2^	ΔAIC
Unspaced	+AoA	4	37.29	30
	+Imageability	4	35.42	28
Spaced	+AoA	4	153.77	146
	+Imageability	4	25.24	17
Unspaced modifier	+AoA	1	4.07	2
	+Imageability	1	3.14	1
Unspaced head	+AoA	1	0.01	1
	+Imageability	1	0.98	1
Spaced modifier	+AoA	1	10.64	9
	+Imageability	1	21.45	20
Spaced head	+AoA	4	25.68	18
	+Imageability	4	12.12	4
VDT	+AoA	4	391.27	383
	+Imageability	4	18.05	10

In Experiment 4, we found that AoA had a significant effect on both accuracy and stimulus duration in a visual duration threshold task. Participants were faster and more accurate at identifying objects they had acquired earlier in life compared with those acquired later in life. These results replicate previous research that has noted an AoA effect during VDT (e.g., Chen et al., 2009; Dent et al., 2007; Preece, 2015). These findings provide evidence in favour of the mapping theory, which is subsumed under the integrated account (Elsherif et al., 2023).

## Comparison between experiments

Although we observed an AoA effect across the tasks, except for the unspaced component identification PDT, the varying size of the AoA effect is likely to differ depending on the number of representations being accessed. As a result, the first confirmatory analysis was to investigate the level of involvement of lexical-semantics in the tasks. Following Elsherif and Catling (2023, 2024), a cross-task comparison was conducted by using combined data together with an additional variable of task. Experiment was included as a fixed effect. To test the unique contribution of each experiment, we used a simple contrast coding scheme. For each contrast, the experiment of interest was coded as −1, while the remaining experiments were coded as 1. This procedure was repeated for each of the experiments in turn, allowing us to obtain the estimated effect for each one relative to the others. When contrast coding is explicitly noted, then there is no requirement to include post hoc testing to determine the directionality of effects, so model coefficients reflect the effect magnitude independently of other predictors’ levels (see Brehm & Alday, 2022, for a detailed discussion of contrast coding in linear mixed models). Given that the datasets were sourced from distinct cohorts, there may be inherent individual differences across samples. However, these groups were matched on a range of demographic characteristics—namely, education, age, gender and racial/ethnic diversity. This methodological consistency facilitates greater external validity and strengthens the generalizability of the results to a broader population. We used a linear mixed model to account for random variance associated with participant and item effects. This allowed us to assess how each of our variables (frequency, imageability, familiarity, and AoA) and their interactions with task influenced the results, consistent with predictions from representation theory.

To assess the interaction between AoA, familiarity, frequency and imageability together with experiment, a combined model was constructed. This model included experiment as an additional fixed factor, in conjunction with the fixed effects of familiarity, imageability and AoA. We also included participant and item as random factors to account for item-level and subject-level variance, respectively. When we analysed stimulus duration using a combined model, we found significant differences between the experiments. PDT with spaced compound words had smaller stimulus duration than PDT with unspaced compound words (*b* = 7.77, *SE* = 3.67,* t* = 2.12) but higher stimulus duration than that of PDT with unspaced component identification (*b* = −10.04, *SE* = 3.66, *t* = −2.74), PDT with spaced component identification (*b* = −11.98, *SE* = 3.66, *t* = −3.27) and visual duration threshold (*b* = −8.87, *SE* = 3.67, *t* = −2.41). Additionally, unspaced compound word PDT had higher stimulus duration than that of PDT with unspaced component identification (*b* = −17.81, *SE* = 3.66, *t* = −4.86), spaced component identification (*b* = −19.75, *SE* = 3.66, *t* = −5.39) and visual duration threshold (*b* = −16.63, *SE* = 3.67, *t* = −4.53). PDT with unspaced component identification had no difference in terms of stimulus duration to that of spaced component identification (*b* = −1.94, *SE* = 3.66,* t* = −0.53) and visual duration (*b* = 1.18, *SE* = 3.67,* t* = 0.32). Visual duration threshold had no difference in terms of stimulus duration to that of PDT with spaced component identification (*b* = −3.12, *SE* = 3.67, *t* = −0.85). The *R*^2^*m* and *R*^2^*c* for combined models was 5.63% and 39.76% respectively. Following this, we applied a likelihood ratio test comparison to compare models with and without the interaction of interest. A series of likelihood ratio tests revealed significant interactions between the experimental task and our key predictor variables. The interaction between experiment and frequency was significant, χ^2^(4) = 704.17, *p* < .001. The interaction between familiarity and experiment was also significant, χ^2^(5) = 162.28, *p* < .001. We also found a significant interaction between experiment and imageability, χ^2^(5) = 446.14, *p* < .001. Finally, the inclusion of an experiment by AoA interaction term resulted in a significant improvement in the model’s fit, χ^2^(5) = 514.33, *p* < .001. The interaction patterns (Fig. 2) showed that all target effects were stronger for the unspaced progressive demasking task than that of the spaced progressive demasking task, VDT and the PDT with component identification tasks.

## Discussion

The study aimed to investigate the AoA effects in the progressive-demasking and visual duration threshold tasks with a large sample of items and participants. Across the tasks, excluding the progressive demasking: component identification for unspaced compound words, we observed an AoA effect of the compound word. When compared with other factors like frequency, familiarity, and imageability across tasks, the strength of the AoA effect aligned with that of semantic factors. This was further supported by the finding that the AoA effect was strongest in tasks that tapped into conceptual and semantic processes, indicating that this task-specific pattern of AoA, suggests a shared origin with lexical-semantic processes. This aligns with the representation theory, which proposes a purely semantic origin for the AoA effect, rather than the mapping or integrated theories.

### Unspaced and spaced compound words in PDT

In PDT for unspaced and spaced compound words, we observed that word frequency, familiarity, AoA and imageability of the compound word influenced identification latency. Here we observed smaller latencies with more frequency, familiarity, imageability, semantic transparency and the earlier the word was acquired. However, we noted that when unspaced compound words were presented, only the AoA of the modifier contributed to the stimulus identification rate, with stimulus duration being earlier for early-acquired modifiers. Our findings for spaced compound words were that the AoA and imageability of the modifier also influenced identification rates, with faster identification rates for earlier-acquired and more imageable modifiers, together with earlier-acquired head lexemes leading to faster identification rates. Based on the interaction between task and AoA, the slope of the AoA effect of the compound word was shallower when presented as a spaced item, compared with when presented unspaced. This demonstrates that the AoA effect is stronger when processing requires direct access to their orthographic–semantic representations, compared with situations where the morphemes are easily separated and their individual meanings are readily accessed.

One possible explanation for the difference in terms of the strength of the AoA effect stems from how readers process spaced and unspaced compound words during PDT. In unspaced compound words (e.g., *airplane*), where the compound is a single unit, the head (e.g., ‘plane’) of the compound word provides a broad semantic category that is further narrowed down by the modifier (e.g., ‘air’). This early access to meaning, because the modifier morpheme is presented separately, could facilitate faster and more accurate recognition of the compound word. Thus, the processing of spaced compound words, as indexed by performance in visual PDT, may rely more on morpheme-based processing, with readers focusing more on the individual meaningful units than on the word as a whole (Benczes, 2005; Elsherif & Catling, 2021).

### The component identification of compound words

In unspaced compound words, our findings suggest that the lexical process may influence the identification of the modifier but not the head. We found that the familiarity of the head only affected the identification of the modifier, while no other predictors influenced the recognition of the head. This is particularly intriguing given the observed AoA effects in other progressive demasking tasks and with spaced compound words. The lack of an AoA effect in unspaced words, despite semantic processing being a necessary but not sufficient component of the AoA effect, suggests a different cognitive strategy is at play. One possible explanation is that the task of identifying a single component from an unspaced compound may force a reliance on a direct lexical route, allowing the system to bypass a full decomposition process. In this scenario, the brain may treat the unspaced compound as a single unit, especially when the target is the head, which is often a standalone word with a strong lexical representation. In contrast, identifying the modifier may be a more complex process that necessitates a deeper search for its related head. This could mean the mapping between the two representations is so systematic and regular that the brain can depend on these established connections between orthography and semantics. Such a predictable mapping requires less time for processing and recognition, reducing the need for semantic properties like AoA and imageability to contribute significantly to identification latencies (Cortese & Schock, 2013). Therefore, we can conclude that the way words are linked to their meaning and other related information changes over time and across different tasks. Thus, our results indicate that lexical-semantic processes change across tasks and word types, underscoring the need for more research into these specific mechanisms and to explain why these effects differ between unspaced and spaced compounds.

In the component identification task for spaced compound words, we observed that the AoA, imageability and name agreement for the compound word affects the component identification task across both positions. Faster identification was associated with earlier acquisition, more imageability and higher name agreement. However, the imageability of the head affects the modifier, while semantic transparency affects the identification of the head. Here, faster identification for the modifier was associated with higher imageability of the head lexeme, whereas faster identification for the head was linked to higher semantic transparency of the compound word. The first position is therefore affected by orthographic–semantics at a lexical and lexemic level, while the head position is affected by orthographic–semantics and relational semantics between the head and the compound word. This indicates that PDT latency for components is primarily influenced by orthographic–semantic processes.

In the VDT task with pictures, we observed that the familiarity, AoA, and imageability of the compound word, along with its name agreement and image agreement, all contributed to the accuracy of the VDT. Specifically, objects that were more familiar, imageable, had higher name and image agreement, and were acquired earlier in life were identified more accurately. In addition, modifiers that were more frequent and imageable, along with those acquired early in life, and heads that were more imageable, led to higher accuracy. However, factors like AoA, imageability, name agreement, image agreement, and visual complexity, as well as the AoA and imageability of the modifier and the imageability of the head, all affected the stimulus duration. These results suggest that lexical-semantic and conceptual processes influence accuracy, while perceptual-semantic processes affect latencies.

The AoA effect influences perceptual processing speed and accuracy during initial recognition. One possible explanation for this stems from how readers process objects. A representation in terms of an organized set of visual features is defined as a known set of object types, which influences the weighting in the system due to higher levels of plasticity (Dent et al., 2007). Our findings indicate that the AoA effect and the modifier effect begin before the conceptual representation, at the perceptual loci. As a result, the AoA effect does not originate at the lexical-semantic level but rather at the perceptual level. These findings are consistent with the multiple loci perspective and mapping theory, especially given that AoA effects have been observed in tasks that assess perceptual processing (Chen et al., 2009; Dent et al., 2007; Preece, 2015). In essence, the AoA effect especially for the morphemic is the result of a perceptual-semantic locus that maps the visual feature to the representations of the individual constituents.

###  Interaction between experiment and AoA

A primary objective of this study was to assess AoA and imageability as drivers of compound word identification. Given the high correlation between these variables (*r* = −0.78), we used nested model comparisons (LRT) and Information Criterion (AIC) to determine their independent contributions. Contrary to the concern that AoA effects might be subsumed by Imageability, our nested models revealed that AoA provides a substantial improvement in model fit for early identification tasks. This pattern suggests that while both factors are theoretically plausible, AoA possesses a unique explanatory power in the mapping between perceptual features and lexical representations that imageability does not fully capture.

Our findings support an Integrated Account of AoA, but with a crucial refinement: The effect appears to accumulate at the interface of perception and semantics where orthographic input is mapped onto semantic representations. In addition, the AoA effects are not localised in the visual system alone but emerge when the task demands a transition from low-level feature extraction to high-level conceptual integration. At this stage, the denser neural connectivity of early-acquired items provides a retrieval advantage. This is evidenced by the absence of a whole-word AoA effect in the unspaced component identification task, when the system focuses strictly on the visual extraction of a sub-unit, it may bypass the deeper perceptual-semantic mapping where AoA resides. In contrast, the robust effects in VDT and spaced PDT suggest that when a complex visual stimulus must be mapped to a single conceptual identity, the 'flexible neural network' (Y.-N. Chang et al., 2019) of early-acquired items facilitates more efficient processing.

This theoretical framework explains why task type significantly interacts with word properties like AoA, frequency, imageability, and familiarity in our data. The findings were contrary to initial expectations. We had predicted that tasks requiring a more holistic visual analysis, such as the spaced progressive demasking task and the VDT with pictures, would show the largest AoA effects. Instead, the greatest effects were observed in unspaced PDT, followed by spaced PDT, with component identification for spaced compound words and VDT with pictures showing the smallest effects. The findings do, however, still support an integrated account of the AoA effect, which postulates that the AoA effect should be larger in tasks more likely to tap into lexical-semantic processes and should be smaller in tasks that do not necessitate semantic processing to the same degree as other tasks (e.g., Catling & Johnston, 2009; see review by Elsherif et al., 2023). This finding also supports the multiple loci theory, which argues that the AoA effect can originate at the perceptual/orthographic level, semantic and phonological level. Here, the integrated account should not begin at the semantic level but between perception and semantic level.

A second important finding was that the AoA effect plateaued in the unspaced component identification task. This suggests that the effect is sensitive to how consistently a word’s spelling maps to its sound. In simpler terms, if the link between spelling and sound is strong and predictable, the AoA effect is reduced. This supports the mapping theory, which posits that early-acquired words create a flexible neural network. Late-acquired words, on the other hand, benefit from this pre-existing structure, so their processing is less influenced by AoA. The PDT with component identification for unspaced compound words ensures that the mapping between representations is regular and systematic; therefore, late-acquired words can benefit from the network structure formed by early-acquired words. These findings suggest that the AoA effect in complex words like compounds is determined by two factors: the strength of the mapping between a word's representations and the gradual development of its perceptual-semantic representations. This aligns with the integrated account of AoA but argues for an earlier starting point for the effect—at the perceptual-semantic stage, not the later lexical-semantic stage as previously thought (Brysbaert & Ellis, 2016; Y.-N. Chang et al., 2019; Dirix & Duyck, 2017a, 2017b; Elsherif et al., 2023).

The mapping theory and the representation theory are often considered complementary, as they explain how and where the AoA effect arises (Dirix & Duyck, 2017a, 2017b). This complementary view provides the theoretical foundation for understanding the different facets of the AoA effect. The representation theory focuses on where the effect resides, positing that it is rooted in the structural organization of our lexical-semantic memory (but see the fact that the AoA effect occurs at the perceptual-semantic level for pictorial stimuli in the current study). The mapping theory focuses on how the effect works, arguing that it results from the efficiency of the connections between different representations. While these theories explain the origin of the effect, they do not fully account for why its magnitude changes across different tasks. This is where the accumulation hypothesis provides a crucial explanation. According to this hypothesis, the AoA effect grows as each additional processing level—such as perception, semantics and phonology—is accessed (Catling & Johnston, 2009; Moore et al., 2004). This theory allows for an integrated account, explaining why the effect becomes stronger in tasks that require more cognitive processing stages. It connects the ‘where’ (representation) and the ‘how’ (mapping) to explain the varying size of the AoA effect across different tasks. This explains why the greatest effects were observed in tasks requiring the most cognitive processing, such as unspaced PDT, followed by spaced PDT, where participants needed to access and integrate lexical and relational semantic and lexical-phonological representations. The smallest effects were seen in PDT with component identification for spaced compound words and VDT with pictures, which require fewer processing stages, primarily lexical semantic processing. The larger effect in these latter tasks highlights the contribution of multiple levels of representation, particularly the greater reliance on semantic processing needed to link a picture or a compound word to its full meaning. Crucially, the absence of an AoA effect for component identification with unspaced compound words suggests that this task primarily relies on low-level visual processing and does not engage the multiple representational levels required for the effect to accumulate. This demonstrates that the AoA effect becomes stronger in tasks that require more cognitive processing stages. To understand the AoA effect more clearly, we need a rigorous, large-scale study that compares different measurement methods. This research should examine how inserting spaces between meaningful units of words affects the processing of compound words and how they relate to object recognition. Specifically, this could be done through a creative destruction approach, a method where researchers pre-define how different, competing theories would explain a complex set of results to see which one holds up best (Tierney et al., 2021).

## Limitations

While our initial analyses utilized a broad set of phonological and physical control variables to account for the noise inherent in demasking paradigms (Ferrand et al., 2011), we have opted for more parsimonious models in this final report. By pruning nonsignificant phonetic predictors (as detailed in our Supplementary Materials), we ensured more stable regression estimates and minimized the risks associated with overparameterisation (Achen, 2005). The fact that our core AoA and imageability effects remained robust across these simplified models increases our confidence in the stability of the reported findings.

A further limitation of the current study concerns the selection of psycholinguistic variables. While our analysis focused on imageability, other factors such as concreteness, valence and arousal are known to influence lexical processing. However, we restricted our scope to imageability because these additional measures were not available for our specific compound stimuli within our norming study. Furthermore, we intentionally avoided using existing large-scale databases for these variables, as many such datasets consist primarily of monomorphemic words or merge monomorphemic and compound ratings (e.g., Scott et al., 2019). Using ratings derived from different morphological structures can ‘contaminate’ the data, as the semantic properties of a compound word are often distinct from its constituent parts (Gagné & Spalding, 2016; Kim et al., 2019; S. Wang et al., 2019). Relying on such merged data would have compromised the internal consistency of our predictors. Consequently, while imageability provides a robust measure of semantic richness, future research should aim to establish comprehensive, compound-specific norms for valence, arousal, and concreteness to further refine models of complex word and object recognition.

The current study captured the full spectrum of semantic transparency by using a naturalistic set of 150 compound words (Janssen et al., 2011), including both transparent (e.g., bedroom) and opaque (e.g., cupboard) items. Future research might further isolate these semantic effects through targeted experimental manipulation. Specifically, extending this paradigm to include 'pseudocompounds' (e.g., ‘carpet’, where constituents are orthographically present but semantically unrelated) would provide a boundary-condition test for the morphological decomposition processes observed here. Such an extension would allow for a more granular comparison between the three theories of AoA, helping to determine at what point the cognitive system ceases to process a word via its constituent mappings and shifts to a purely holistic representation. Combining natural language stimuli with these manipulated forms will be essential for a comprehensive model of how morphological history influences both lexical and visual identification.

Regarding our VDT paradigm, we observed that approximately 52% of observations occurred at the 50-ms floor. While our analysis confirms that this floor provided sufficient variance to detect significant AoA and morphemic effects, future studies using higher-specification hardware or more controlled lab settings should consider a shorter initial duration (e.g., 16.67 ms or 33.33 ms). This would allow for an even more granular distribution of identification thresholds at the faster end of the processing spectrum. Nevertheless, our choice of a 50 ms baseline served as a necessary technical 'buffer' to ensure data integrity across the varied hardware latencies inherent in our sample, and the robustness of our AoA findings suggests that this pragmatic constraint did not mask the underlying lexical signals.

A final limitation was that all 150 compound stimuli represented physical objects, meaning the dataset was composed entirely of concrete words. This selection was necessary to facilitate the comparison between word processing and object recognition tasks. Consequently, concreteness was held constant across the stimuli set to avoid ceiling effects in the norming data. By maintaining this high level of concreteness, we were able to isolate the effects of imageability without the confounding influence of the abstract-concrete distinction. This control ensures that any observed differences are task-specific, allowing for a clearer assessment of the underlying cognitive processes. Our findings suggest that participants primarily utilized meaning-based predictors to identify compound words or objects, regardless of whether the task required access to lexical representations, as influenced by the presence or absence of spacing between lexemes. Given that 'the sample [size] of each experiment was moderate and the designs were also slightly different' (Elsherif et al., 2017, p. 26), these observed variations should be still interpreted with caution.

## Conclusion

Building on previous research that AoA effects are present in monomorphemic words and production tasks (e.g., Ellis & Morrison, 1998), the present study confirms that a similar pattern of findings are observed for compound words, using written and pictorial stimuli. Our findings lead us to propose that the AoA effect begins at the perceptual-semantic level, a stage that matures over time. This effect arises from the inconsistent mapping between different representations (e.g., spelling and meaning). This proposed origin aligns with the integrated account of the AoA effect, indicating that the AoA effect starts earlier than previously thought, between perceptual and conceptual/semantics loci and at the irregular mapping between representations. In addition, the integrated account requires the accumulation hypothesis to be included to explain the varying levels of the AoA effect.

## Supplementary Information

Below is the link to the electronic supplementary material.

Supplementary file1 (DOCX 116 KB)

## Data Availability

All data are publicly available at: https://osf.io/knwur/. Research materials will be made available via the Gorilla Open Materials repository: https://app.gorilla.sc/openmaterials/1106906.

## Footnotes

1 Westfall et al. (2014) published a theoretical analysis of a mixed-effects model and created a website (https://jakewestfall.shinyapps.io/two_factor_power/), which allows researchers to calculate the power of an experiment and the number of items/participants required for a well-powered experiment. We estimated the sample size using the following values: Effect size *d*: 0.063, residual variance: 0.61, participant intercept variance: 0.23, item intercept variance: 0.16, participant-by-item variance: 0.219, participant slope variance: 0, item slope variance: 0.

2 To be in line with Open Scholarship principles, the original 'full' models containing the complete set of phonetic features are provided in the Supplementary Materials (Tables S4–S7) to maintain full transparency and alignment with our shared data and code.

## Code availability

All code is publicly available at: https://osf.io/knwur/
