# Supplementary material for: Age-of-acquisition affects object recognition and compound word identification: Evidence from visual duration thresholds and progressive demasking
Source: Atten Percept Psychophys. 2026 May 13;88(5):129. doi: 10.3758/s13414-026-03267-y (PMC13171731; doi:10.3758/s13414-026-03267-y)
Supplement: Supplementary file 1 — Supplementary file1 (DOCX 116 KB) [file 13414_2026_3267_MOESM1_ESM.docx]

**Table S1.**

*The baseline model results for Reaction time of correct responses in the progressive demasking task*

| Effect |  | | Stimulus duration | | | |  | |  | |
| --- | --- | --- | --- | --- | --- | --- | --- | --- | --- | --- |
|  |  | *β* | | *SE* | *2.5%* | *97.5%* | *T* | *R2m* | | *R2c* |
| *Experiment 1: Unspaced compound word PDT^a^* |  |  | |  |  |  |  |  | |  |
| L |  | **502.75** | | **42.70** | **419.06** | **586.44** | **11.77** | **0.03** | | **0.137** |
| CFreq |  | -42.10 | | 41.22 | -122.89 | 38.69 | -1.02 | 0.03 | | 0.137 |
| MFreq |  | -21.81 | | 39.41 | -99.05 | 55.43 | -0.55 | 0.03 | | 0.137 |
| HFreq |  | -76.58 | | 38.93 | -152.88 | -0.28 | -1.97 | 0.03 | | 0.137 |
| bilabial |  | -89.57 | | 72.70 | -232.06 | 52.92 | -1.23 | 0.03 | | 0.137 |
| labiodental |  | -59.69 | | 93.74 | -243.42 | 124.04 | -0.64 | 0.03 | | 0.137 |
| dental |  | -144.25 | | 238.24 | -611.2 | 322.7 | -0.61 | 0.03 | | 0.137 |
| labiovelar |  | -87.02 | | 84.82 | -253.27 | 79.23 | -1.03 | 0.03 | | 0.137 |
| alveolar |  | -48.53 | | 76.22 | -197.92 | 100.86 | -0.64 | 0.03 | | 0.137 |
| Palatal.alveolar |  | -87.10 | | 123.04 | -328.26 | 154.06 | -0.71 | 0.03 | | 0.137 |
| Glottal |  | -33.42 | | 96.68 | -222.91 | 156.07 | -0.35 | 0.03 | | 0.137 |
| velar |  | 91.06 | | 95.58 | -96.28 | 278.4 | 0.95 | 0.03 | | 0.137 |
| voiced |  | **159.14** | | **46.15** | **68.69** | **249.59** | **3.45** | **0.03** | | **0.137** |
|  |  |  | |  |  |  |  |  | |  |
| *Experiment 2: Spaced compound word PDT^a^* |  |  | |  |  |  |  |  | |  |
| L |  | **375.90** | | **26.43** | **324.1** | **427.7** | **14.22** | **0.046** | | **0.230** |
| CFreq |  | -19.25 | | 25.64 | -69.5 | 31 | -0.75 | 0.046 | | 0.230 |
| MFreq |  | -29.20 | | 24.43 | -77.08 | 18.68 | -1.20 | 0.046 | | 0.230 |
| HFreq |  | -3.778 | | 24.32 | -51.45 | 43.89 | -0.16 | 0.046 | | 0.230 |
| bilabial |  | 58.34 | | 45.20 | -30.25 | 146.93 | 1.29 | 0.046 | | 0.230 |
| labiodental |  | -86.18 | | 58.00 | -199.86 | 27.5 | -1.49 | 0.046 | | 0.230 |
| dental |  | 120.82 | | 145.89 | -165.12 | 406.76 | 0.83 | 0.046 | | 0.230 |
| labiovelar |  | -47.37 | | 52.81 | -150.88 | 56.14 | -0.90 | 0.046 | | 0.230 |
| alveolar |  | -25.29 | | 47.38 | -118.15 | 67.57 | -0.54 | 0.046 | | 0.230 |
| Palatal.alveolar |  | -36.15 | | 76.25 | -185.6 | 113.3 | -0.47 | 0.046 | | 0.230 |
| Glottal |  | -23.66 | | 60.28 | -141.81 | 94.49 | -0.39 | 0.046 | | 0.230 |
| velar |  | -38.06 | | 59.53 | -154.74 | 78.62 | 0.64 | 0.046 | | 0.230 |
| voiced |  | -15.91 | | 28.65 | -72.06 | 40.24 | -0.56 | 0.046 | | 0.230 |
|  |  |  | |  |  |  |  |  | |  |
| *Experiment 3a: Unspaced compound word- Identification of the Modifier lexeme ^a^* |  |  | |  |  |  |  |  | |  |
| L |  | 81.05 | | 49.88 | -16.71 | 178.81 | 1.63 | 0.002 | | 0.070 |
| CFreq |  | 52.19 | | 47.72 | -41.34 | 145.72 | 1.09 | 0.002 | | 0.070 |
| MFreq |  | **101.31** | | **45.92** | **11.31** | **191.31** | **2.21*** | **0.002** | | **0.070** |
| HFreq |  | 83.40 | | 46.07 | -6.9 | 173.7 | 1.81 | 0.002 | | 0.070 |
| bilabial |  | -40.70 | | 85.72 | -208.71 | 127.31 | -0.48 | 0.002 | | 0.070 |
| labiodental |  | -61.75 | | 108.83 | -275.06 | 151.56 | -0.57 | 0.002 | | 0.070 |
| dental |  | 114.50 | | 284.52 | -443.16 | 672.16 | 0.40 | 0.002 | | 0.070 |
| labiovelar |  | -37.93 | | 99.51 | -232.97 | 157.11 | -0.38 | 0.002 | | 0.070 |
| alveolar |  | 9.475 | | 89.35 | -165.65 | 184.6 | 0.11 | 0.002 | | 0.070 |
| Palatal.alveolar |  | -164.55 | | 139.23 | -437.44 | 108.34 | -1.18 | 0.002 | | 0.070 |
| Glottal |  | 111.67 | | 113.36 | -110.52 | 333.86 | 0.99 | 0.002 | | 0.070 |
| velar |  | -55.30 | | 113.40 | -277.56 | 166.96 | -0.49 | 0.002 | | 0.070 |
| voiced |  | 22.62 | | 53.96 | -83.14 | 128.38 | 0.42 | 0.002 | | 0.070 |
| *Experiment 3a: Unspaced compound word- Identification of the Head lexeme ^a^* |  |  | |  |  |  |  |  | |  |
| L |  | -48.31 | | 46.80 | -140.04 | 43.42 | 0.42 | 0.001 | | 0.087 |
| CFreq |  | -14.60 | | 45.85 | -104.47 | 75.27 | 0.19 | 0.001 | | 0.087 |
| MFreq |  | -26.01 | | 43.37 | -111.02 | 59 | 1.43 | 0.001 | | 0.087 |
| HFreq |  | -28.91 | | 42.78 | -112.76 | 54.94 | 0.72 | 0.001 | | 0.087 |
| bilabial |  | 93.35 | | 79.53 | -62.53 | 249.23 | -0.63 | 0.001 | | 0.087 |
| labiodental |  | 45.53 | | 102.91 | -156.17 | 247.23 | 0.82 | 0.001 | | 0.087 |
| dental |  | 479.61 | | 248.53 | -7.51 | 966.73 | 0.42 | 0.001 | | 0.087 |
| labiovelar |  | 32.24 | | 93.06 | -150.16 | 214.64 | -0.36 | 0.001 | | 0.087 |
| alveolar |  | 62.16 | | 83.54 | -101.58 | 225.9 | -1.23 | 0.001 | | 0.087 |
| Palatal.alveolar |  | 64.11 | | 140.94 | -212.13 | 340.35 | -0.12 | 0.001 | | 0.087 |
| Glottal |  | 42.99 | | 106.34 | -165.44 | 251.42 | -0.15 | 0.001 | | 0.087 |
| velar |  | 82.04 | | 103.90 | -121.6 | 285.68 | 0.61 | 0.001 | | 0.087 |
| voiced |  | -3.555 | | 50.71 | -102.95 | 95.84 | 0.34 | 0.001 | | 0.087 |
| *Experiment 3b: Spaced compound word –Identification of the Modifier lexeme ^a^* |  |  | |  |  |  |  |  | |  |
| L |  | **173.13** | | **54.74** | **65.84** | **280.42** | **3.16*** | **0.003** | | **0.047** |
| CFreq |  | 2.917 | | 53.13 | -101.22 | 107.05 | 0.06 | 0.003 | | 0.047 |
| MFreq |  | 20.38 | | 50.64 | -78.87 | 119.63 | 0.40 | 0.003 | | 0.047 |
| HFreq |  | -24.36 | | 50.35 | -123.05 | 74.33 | -0.48 | 0.003 | | 0.047 |
| bilabial |  | -167.79 | | 94.00 | -352.03 | 16.45 | -1.79 | 0.003 | | 0.047 |
| labiodental |  | -194.85 | | 120.29 | -430.62 | 40.92 | -1.62 | 0.003 | | 0.047 |
| dental |  | -86.21 | | 306.89 | -687.71 | 515.29 | -0.28 | 0.003 | | 0.047 |
| labiovelar |  | **-332.49** | | **109.64** | **-547.38** | **-117.6** | **-3.03*** | **0.003** | | **0.047** |
| alveolar |  | **-262.85** | | **98.39** | **-455.69** | **-70.01** | **-2.67*** | **0.003** | | **0.047** |
| Palatal.alveolar |  | -275.81 | | 158.46 | -586.39 | 34.77 | -1.74 | 0.003 | | 0.047 |
| Glottal |  | -173.09 | | 124.78 | -417.66 | 71.48 | -1.39 | 0.003 | | 0.047 |
| velar |  | -184.002 | | 123.25 | -425.57 | 57.57 | -1.49 | 0.003 | | 0.047 |
| voiced |  | 30.87 | | 59.41 | -85.57 | 147.31 | 0.52 | 0.003 | | 0.047 |
| *Experiment 3b: Spaced compound word - Identification of the Head lexeme ^a^* |  |  | |  |  |  |  |  | |  |
| L |  | **294.07** | | **44.76** | **206.34** | **381.8** | **6.57*** | **0.014** | | **0.115** |
| CFreq |  | 9.209 | | 43.38 | -75.82 | 94.23 | 0.21 | 0.014 | | 0.115 |
| MFreq |  | -63.83 | | 41.28 | -144.74 | 17.08 | -1.55 | 0.014 | | 0.115 |
| HFreq |  | 28.46 | | 41.04 | -51.98 | 108.9 | 0.69 | 0.014 | | 0.115 |
| bilabial |  | 149.47 | | 76.46 | -0.39 | 299.33 | 1.96 | 0.014 | | 0.115 |
| labiodental |  | 151.64 | | 97.96 | -40.36 | 343.64 | 1.55 | 0.014 | | 0.115 |
| dental |  | 436.62 | | 247.43 | -48.34 | 921.58 | 1.77 | 0.014 | | 0.115 |
| labiovelar |  | 39.569 | | 89.30 | -135.46 | 214.6 | 0.44 | 0.014 | | 0.115 |
| alveolar |  | 188.01 | | 80.08 | 31.05 | 344.97 | 2.35 | 0.014 | | 0.115 |
| Palatal.alveolar |  | 96.53 | | 128.82 | -155.96 | 349.02 | 0.75 | 0.014 | | 0.115 |
| Glottal |  | 110.990 | | 101.84 | -88.62 | 310.6 | 1.09 | 0.014 | | 0.115 |
| velar |  | 160.67 | | 100.49 | -36.29 | 357.63 | 1.60 | 0.014 | | 0.115 |
| voiced |  | **131.79** | | **48.38** | **36.97** | **226.61** | **2.72*** | **0.014** | | **0.115** |

|  |
| --- |
|  |

*Note***.** orthographic length (L) and frequency (CFreq) of the compound word, word frequency of the modifier (MFreq) and head (HFreq). SE = Standard error. *Significant at the *p* = .05 level after Holm-Bonferroni method and bolded. ^a^ This model converged only with a random subject and item intercept and no predictor was included as part of a random slope.

**Table S2.**

*The baseline model + predictors for reaction time of correct responses in the progressive demasking task*

| Effect |  |  | Stimulus duration | | | | | |
| --- | --- | --- | --- | --- | --- | --- | --- | --- |
|  | *Β* | | *SE* | *2.5%* | *97.5%* | *T* | *R2m* | *R2c* |
| *Experiment 1: Unspaced compound word PDT^a^* |  | |  |  |  |  |  |  |
| CFam | 43.26 | | 38.96 | -33.1 | 119.62 | 1.11 | 0.033 | 0.137 |
| CAoA | 47.47 | | 44.24 | -39.24 | 134.18 | 1.07 | 0.033 | 0.137 |
| CI | -21.11 | | 42.16 | -103.74 | 61.52 | -0.50 | 0.033 | 0.137 |
| ST | -0.342 | | 40.20 | -79.13 | 78.45 | -0.01 | 0.033 | 0.137 |
| LMD | **-93.97** | | **37.58** | **-167.63** | **-20.31** | **-2.50^*^** | **0.034** | **0.137** |
| NA (H) | 72.00 | | 39.31 | -5.05 | 149.05 | 1.83 | 0.033 | 0.137 |
| NA (%) | -52.48 | | 39.55 | -130 | 25.04 | -1.33 | 0.033 | 0.137 |
| IA | 13.83 | | 38.48 | -61.59 | 89.25 | 0.36 | 0.033 | 0.137 |
| VC | 8.700 | | 38.84 | -67.43 | 84.83 | 0.22 | 0.033 | 0.137 |
| MFam | -9.100 | | 52.18 | -111.37 | 93.17 | -0.17 | 0.033 | 0.137 |
| MAoA | 48.91 | | 47.18 | -43.56 | 141.38 | 1.04 | 0.033 | 0.137 |
| MI | -17.43 | | 38.95 | -93.77 | 58.91 | -0.45 | 0.033 | 0.137 |
| Hfam | 37.35 | | 48.33 | -57.38 | 132.08 | 0.77 | 0.033 | 0.137 |
| HAoA | -8.017 | | 45.33 | -96.86 | 80.83 | -0.18 | 0.033 | 0.137 |
| HI | 57.94 | | 39.37 | -19.23 | 135.11 | 1.47 | 0.033 | 0.137 |
|  |  | |  |  |  |  |  |  |
| *Experiment 2: Spaced compound word PDT^b^* |  | |  |  |  |  |  |  |
| CFam | 3.319 | | 24.37 | -44.45 | 51.08 | 0.14 | 0.046 | 0.230 |
| CAoA | 7.333 | | 27.64 | -46.84 | 61.51 | 0.27 | 0.046 | 0.230 |
| CI | -2.516 | | 26.21 | -53.89 | 48.86 | -0.10 | 0.046 | 0.230 |
| ST | -19.51 | | 24.94 | -68.39 | 29.37 | -0.78 | 0.046 | 0.230 |
| LMD | -27.79 | | 23.88 | -74.59 | 19.01 | -1.16 | 0.046 | 0.230 |
| NA (H) | 30.88 | | 24.58 | -17.3 | 79.06 | 1.26 | 0.046 | 0.230 |
| NA (%) | -28.44 | | 24.60 | -76.66 | 19.78 | -1.16 | 0.046 | 0.230 |
| IA | -10.22 | | 24.02 | -57.3 | 36.86 | -0.43 | 0.046 | 0.230 |
| VC | -10.40 | | 24.14 | -57.71 | 36.91 | -0.43 | 0.046 | 0.230 |
| MFam | 34.41 | | 32.41 | -29.11 | 97.93 | 1.06 | 0.046 | 0.230 |
| MAoA | 15.75 | | 29.42 | -41.91 | 73.41 | 0.54 | 0.046 | 0.230 |
| MI | -21.05 | | 24.08 | -68.25 | 26.15 | -0.87 | 0.046 | 0.230 |
| Hfam | 8.638 | | 30.06 | -50.27 | 67.55 | 0.29 | 0.046 | 0.230 |
| HAoA | -8.655 | | 28.14 | -63.81 | 46.5 | -0.31 | 0.046 | 0.230 |
| HI | -14.05 | | 24.67 | -62.4 | 34.3 | -0.57 | 0.046 | 0.230 |
|  |  | |  |  |  |  |  |  |
| *Experiment 3a: Unspaced compound word- Identification of the Modifier lexeme ^a^* |  | |  |  |  |  |  |  |
| CFam | -8.153 | | 46.26 | -98.82 | 82.52 | -0.18 | 0.002 | 0.070 |
| CAoA | 78.30 | | 52.27 | -24.15 | 180.75 | 1.50 | 0.002 | 0.070 |
| CI | -2.637 | | 49.60 | -99.85 | 94.58 | -0.05 | 0.002 | 0.070 |
| ST | 20.862 | | 47.09 | -71.43 | 113.16 | 0.44 | 0.002 | 0.070 |
| LMD | -2.567 | | 44.46 | -89.71 | 84.57 | -0.06 | 0.002 | 0.070 |
| NA (H) | -33.89 | | 46.43 | -124.89 | 57.11 | -0.73 | 0.002 | 0.070 |
| NA (%) | 20.82 | | 46.12 | -69.58 | 111.22 | 0.45 | 0.002 | 0.070 |
| IA | 7.666 | | 45.20 | -80.93 | 96.26 | 0.17 | 0.002 | 0.070 |
| VC | 40.93 | | 45.21 | -47.68 | 129.54 | 0.91 | 0.002 | 0.070 |
| MFam | 41.17 | | 61.04 | -78.47 | 160.81 | 0.68 | 0.002 | 0.070 |
| MAoA | 0.756 | | 55.13 | -107.3 | 108.81 | 0.01 | 0.002 | 0.070 |
| MI | 17.46 | | 44.99 | -70.72 | 105.64 | 0.39 | 0.002 | 0.070 |
| Hfam | 76.24 | | 55.96 | -33.44 | 185.92 | 1.36 | 0.002 | 0.070 |
| HAoA | 22.67 | | 52.37 | -79.98 | 125.32 | 0.43 | 0.002 | 0.070 |
| HI | -19.30 | | 46.06 | -109.58 | 70.98 | -0.42 | 0.002 | 0.070 |
|  |  | |  |  |  |  |  |  |
| *Experiment 3a: Unspaced compound word- Identification of the Head lexeme ^a^* |  | |  |  |  |  |  |  |
| CFam | 43.53 | | 42.65 | -40.06 | 127.12 | 1.02 | 0.001 | 0.087 |
| CAoA | **-114.03** | | **48.45** | **-208.99** | **-19.07** | **-2.35*** | **0.002** | **0.088** |
| CI | 79.86 | | 46.06 | -10.42 | 170.14 | 1.73 | 0.001 | 0.087 |
| ST | 36.92 | | 43.88 | -49.08 | 122.92 | 0.84 | 0.001 | 0.087 |
| LMD | -13.96 | | 42.81 | -97.87 | 69.95 | -0.33 | 0.001 | 0.087 |
| NA (H) | -60.37 | | 43.49 | -145.61 | 24.87 | -1.39 | 0.001 | 0.087 |
| NA (%) | 45.83 | | 43.94 | -40.29 | 131.95 | 1.04 | 0.001 | 0.087 |
| IA | -4.70 | | 42.44 | -87.88 | 78.48 | -0.11 | 0.001 | 0.087 |
| VC | -61.12 | | 42.48 | -144.38 | 22.14 | -1.44 | 0.001 | 0.087 |
| MFam | -9.738 | | 57.48 | -122.4 | 102.92 | -0.17 | 0.001 | 0.087 |
| MAoA | -64.32 | | 51.97 | -166.18 | 37.54 | -1.24 | 0.001 | 0.087 |
| MI | 11.18 | | 43.05 | -73.2 | 95.56 | 0.26 | 0.001 | 0.087 |
| Hfam | 50.49 | | 53.47 | -54.31 | 155.29 | 0.94 | 0.001 | 0.087 |
| HAoA | -116.40 | | 50.16 | -214.71 | -18.09 | -2.32 | 0.002 | 0.088 |
| HI | 22.91 | | 2.91 | 17.21 | 28.61 | 0.52 | 0.001 | 0.087 |
|  |  | |  |  |  |  |  |  |
| *Experiment 3b: Spaced compound word –Identification of the Modifier lexeme ^a^* |  | |  |  |  |  |  |  |
| CFam | -15.97 | | 50.52 | -114.99 | 83.05 | -0.32 | 0.003 | 0.047 |
| CAoA | 33.96 | | 57.90 | -79.52 | 147.44 | 0.59 | 0.003 | 0.047 |
| CI | 3.848 | | 54.47 | -102.91 | 110.61 | 0.07 | 0.003 | 0.047 |
| ST | -24.26 | | 51.81 | -125.81 | 77.29 | -0.47 | 0.003 | 0.047 |
| LMD | -40.71 | | 49.62 | -137.97 | 56.55 | -0.82 | 0.003 | 0.047 |
| NA (H) | -36.56 | | 51.09 | -136.7 | 63.58 | -0.72 | 0.003 | 0.047 |
| NA (%) | -43.73 | | 51.05 | -143.79 | 56.33 | -0.86 | 0.003 | 0.047 |
| IA | **-127.02** | | **48.66** | **-222.39** | **-31.65** | **-2.61*** | **0.004** | **0.047** |
| VC | 43.93 | | 49.82 | -53.72 | 141.58 | 0.88 | 0.003 | 0.047 |
| MFam | 57.74 | | 67.21 | -73.99 | 189.47 | 0.86 | 0.003 | 0.047 |
| MAoA | -30.98 | | 60.96 | -150.46 | 88.5 | -0.51 | 0.003 | 0.047 |
| MI | -46.04 | | 49.89 | -143.82 | 51.74 | -0.92 | 0.003 | 0.047 |
| Hfam | -54.92 | | 62.14 | -176.71 | 66.87 | -0.88 | 0.003 | 0.047 |
| HAoA | 47.31 | | 58.14 | -66.64 | 161.26 | 0.81 | 0.003 | 0.047 |
| HI | -62.355 | | 50.87 | -162.06 | 37.35 | -1.23 | 0.003 | 0.047 |
|  |  | |  |  |  |  |  |  |
| *Experiment 3b: Spaced compound word –Identification of the Head lexeme ^a^* |  | |  |  |  |  |  |  |
| CFam | -24.07 | | 41.17 | -104.76 | 56.62 | -0.59 | 0.014 | 0.115 |
| CAoA | 81.78 | | 46.20 | -8.77 | 172.33 | 1.77 | 0.014 | 0.115 |
| CI | -46.39 | | 44.20 | -133.02 | 40.24 | -1.05 | 0.014 | 0.115 |
| ST | -88.22 | | 41.61 | -169.78 | -6.66 | -2.12 | 0.015 | 0.115 |
| LMD | -6.252 | | 40.57 | -85.77 | 73.27 | -0.15 | 0.014 | 0.115 |
| NA (H) | 82.62 | | 41.19 | 1.89 | 163.35 | 2.01 | 0.015 | 0.115 |
| NA (%) | -64.72 | | 41.44 | -145.94 | 16.5 | -1.56 | 0.014 | 0.115 |
| IA | -21.95 | | 40.58 | -101.49 | 57.59 | -0.54 | 0.014 | 0.115 |
| VC | 46.65 | | 40.60 | -32.93 | 126.23 | 1.15 | 0.014 | 0.115 |
| MFam | -64.38 | | 54.75 | -171.69 | 42.93 | -1.18 | 0.014 | 0.115 |
| MAoA | 60.33 | | 49.50 | -36.69 | 157.35 | 1.22 | 0.014 | 0.115 |
| MI | **-99.65** | | **39.89** | **-177.83** | **-21.47** | **-2.50*** | **0.015** | **0.115** |
| Hfam | 54.88 | | 50.57 | -44.24 | 154 | 1.09 | 0.014 | 0.115 |
| HAoA | -29.41 | | 47.51 | -122.53 | 63.71 | -0.62 | 0.014 | 0.115 |
| HI | -67.86 | | 41.22 | -148.65 | 12.93 | -1.65 | 0.014 | 0.115 |

*Note***.** Conceptual familiarity of the compound word (CFam), age of acquisition of the compound word (CAoA), imageability of the compound word (CI), semantic transparency (ST), Lexeme meaning dominance (LMD), name agreement: H index (NA(H)) and percent (%), image agreement (IA) visual complexity (VC) of the compound word, familiarity of the modifier lexeme (MFam), Age of acquisition of the modifier lexeme (MAoA) and imageability of the modifier lexeme (MI). Familiarity of the head lexeme (Hfam), age of acquisition of the head lexeme (HAoA) and imageability of the head lexeme (HI). SE = Standard error. *Significant at the *p* = .05 level after Holm-Bonferroni method and bolded. ^a^ This model converged only with a random subject and item intercept and no predictor was included as part of a random slope ^b^ This model only converged for RT of correct responses when a random subject-intercept model is included

**Table S3.**

*Sensitivity Analysis of the baseline model and also predictors for stimulus duration threshold of correct responses in the visual duration task (Excluding 50ms Floor Data)*

| Effect |  | | Stimulus duration | | | |  | |  |
| --- | --- | --- | --- | --- | --- | --- | --- | --- | --- |
|  |  | *β* | | *SE* | *2.5%* | *97.5%* | *T* | *R2m* | *R2c* |
| L^b^ |  | -0.221 | | 0.665 | -1.524 | 1.082 | -0.33 | 0.03 | 0.282 |
| CFreq^b^ |  | **-1.919** | | **0.652** | **-3.197** | **-0.641** | **-2.95** | **0.03** | **0.282** |
| MFreq^b^ |  | 0.185 | | 0.613 | -1.016 | 1.386 | 0.30 | 0.03 | 0.282 |
| HFreq^b^ |  | -1.168 | | 0.613 | -2.369 | 0.033 | -1.91 | 0.03 | 0.282 |
| bilabial^b^ |  | 1.146 | | 1.176 | -1.159 | 3.451 | 0.97 | 0.03 | 0.282 |
| labiodental^b^ |  | -0.324 | | 1.489 | -3.242 | 2.594 | -0.22 | 0.03 | 0.282 |
| dental^b^ |  | -1.524 | | 3.653 | -8.684 | 5.636 | -0.42 | 0.03 | 0.282 |
| Labiovelar^b^ |  | -0.643 | | 1.342 | -3.273 | 1.987 | 0.48 | 0.03 | 0.282 |
| alveolar^b^ |  | 0.373 | | 1.238 | -2.053 | 2.799 | 0.30 | 0.03 | 0.282 |
| Palatal.alveolar^b^ |  | 1.910 | | 1.966 | -1.943 | 5.763 | 0.97 | 0.03 | 0.282 |
| Glottal^b^ |  | 1.416 | | 1.560 | -1.642 | 4.474 | 0.91 | 0.03 | 0.282 |
| velar^b^ |  | 1.521 | | 1.530 | -1.478 | 4.52 | 0.99 | 0.03 | 0.282 |
| Voiced^b^ |  | 0.103 | | 0.729 | -1.326 | 1.532 | 0.14 | 0.03 | 0.282 |
| CFam^a^ |  | -1.047 | | 0.658 | -2.337 | 0.243 | -1.59 | 0.03 | 0.290 |
| CAoA^a^ |  | **5.687** | | **1.065** | **3.6** | **7.774** | **5.34** | **0.063** | **0.308** |
| CI^a^ |  | **-3.127** | | **0.691** | **-4.481** | **-1.773** | **-4.53** | **0.056** | **0.289** |
| ST^b^ |  | -1.383 | | 0.638 | -2.633 | -0.133 | -2.17 | 0.036 | 0.283 |
| LMD^b^ |  | -0.624 | | 0.617 | -1.833 | 0.585 | -1.01 | 0.030 | 0.282 |
| NA (H)^b^ |  | **2.202** | | **0.602** | **1.022** | **3.382** | **3.66** | **0.048** | **0.284** |
| NA (%)^b^ |  | **-2.832** | | **0.859** | **-4.516** | **-1.148** | **-3.30** | **0.040** | **0.285** |
| IA^a^ |  | **-2.666** | | **0.562** | **-3.768** | **-1.564** | **-4.74** | **0.057** | **0.283** |
| VC^a^ |  | 1.373 | | 0.639 | 0.121 | 2.625 | 2.15 | 0.037 | 0.289 |
| MFam^b^ |  | -0.082 | | 0.846 | -1.74 | 1.576 | -0.10 | 0.029 | 0.283 |
| MAoA^a^ |  | **3.659** | | **0.979** | **1.74** | **5.578** | **3.74** | **0.045** | **0.291** |
| MI^b^ |  | **-1.667** | | **0.589** | **-2.821** | **-0.513** | **-2.83** | **0.043** | **0.284** |
| Hfam^b^ |  | -0.087 | | 0.764 | -1.584 | 1.41 | -0.11 | 0.029 | 0.283 |
| HAoA^a^ |  | 1.254 | | 0.709 | -0.136 | 2.644 | 1.77 | 0.034 | 0.283 |
| HI^b^ |  | **-1.406** | | **0.611** | **-2.604** | **-0.208** | **-2.30** | **0.037** | **0.282** |

*Note***.** Orthographic length (L) and frequency (CFreq) of the compound word, word frequency of the modifier (MFreq) and head (HFreq). Conceptual familiarity of the compound word (CFam), age of acquisition of the compound word (CAoA), imageability of the compound word (CI), semantic transparency (ST), Lexeme meaning dominance (LMD), name agreement: H index (NA(H)) and percent (%), image agreement (IA) visual complexity (VC) of the compound word, familiarity of the modifier lexeme (MFam), Age of acquisition of the modifier lexeme (MAoA) and imageability of the modifier lexeme (MI). Familiarity of the head lexeme (Hfam), age of acquisition of the head lexeme (HAoA) and imageability of the head lexeme (HI). SE = Standard error. *Significant at the *p* = .05 level after Holm-Bonferroni method and bolded. ^a^ This model converged only with a random subject and item intercept and no predictor was included as part of a random slope ^b^ This model only converged for RT of correct responses when a random subject-intercept model is included

**Table S4.**

*The baseline model results for stimulus duration of correct responses in the progressive demasking task with phonetic complexity*

| Effect |  | | Stimulus duration | | | |  | |  | |
| --- | --- | --- | --- | --- | --- | --- | --- | --- | --- | --- |
|  |  | *β* | | *SE* | *2.5%* | *97.5%* | *T* | *R2m* | | *R2c* |
| *Experiment 1: Unspaced compound word PDT^a^* |  |  | |  |  |  |  |  | |  |
| L |  | 0.034 | | 1.311 | -2.536 | 2.604 | 0.26 | 0.08 | | 0.420 |
| CFreq |  | **-9.346** | | **1.271** | **-11.84** | **-6.855** | **-7.36*** | **0.08** | | **0.420** |
| MFreq |  | 0.654 | | 1.210 | -1.718 | 3.026 | -0.54 | 0.08 | | 0.420 |
| HFreq |  | -2.156 | | 1.203 | -4.514 | 0.202 | -1.79 | 0.08 | | 0.420 |
| bilabial |  | 4.424 | | 2.220 | 0.073 | 8.775 | 1.99 | 0.08 | | 0.420 |
| labiodental |  | 3.797 | | 2.867 | -1.822 | 9.416 | 1.32 | 0.08 | | 0.420 |
| dental |  | 7.540 | | 2.483 | 2.673 | 12.41 | 1.04 | 0.08 | | 0.420 |
| labiovelar |  | 4.824 | | 2.607 | -0.285 | 9.934 | 1.85 | 0.08 | | 0.420 |
| alveolar |  | 1.854 | | 2.338 | -2.728 | 6.436 | 0.79 | 0.08 | | 0.420 |
| Palatal.alveolar |  | 2.566 | | 3.777 | -4.837 | 9.969 | 0.68 | 0.08 | | 0.420 |
| Glottal |  | 4.616 | | 2.978 | -1.221 | 10.45 | 1.55 | 0.08 | | 0.420 |
| velar |  | 4.553 | | 2.941 | -1.211 | 10.32 | 1.55 | 0.08 | | 0.420 |
| voiced |  | -1.426 | | 1.421 | -4.211 | 1.359 | -1.00 | 0.08 | | 0.420 |
|  |  |  | |  |  |  |  |  | |  |
| *Experiment 2: Spaced compound word PDT^a^* |  |  | |  |  |  |  |  | |  |
| L |  | 0.011 | | 0.072 | -0.130 | 0.152 | -0.15 | 0.010 | | 0.508 |
| CFreq |  | **-1.620** | | **0.069** | **-1.755** | **-1.485** | **-2.34*** | **0.010** | | **0.508** |
| MFreq |  | -0.031 | | 0.066 | -0.160 | 0.098 | -0.05 | 0.010 | | 0.508 |
| HFreq |  | -0.316 | | 0.657 | -1.604 | 0.972 | -0.48 | 0.010 | | 0.508 |
| bilabial |  | **2.839** | | **1.219** | **0.450** | **5.228** | **2.33*** | **0.010** | | **0.508** |
| labiodental |  | 2.624 | | 1.567 | -0.447 | 5.695 | 1.68 | 0.010 | | 0.508 |
| dental |  | 5.554 | | 3.949 | -2.186 | 13.29 | 1.41 | 0.010 | | 0.508 |
| labiovelar |  | 2.478 | | 1.423 | -0.311 | 5.267 | 1.74 | 0.010 | | 0.508 |
| alveolar |  | 0.240 | | 1.280 | -2.269 | 2.749 | 0.19 | 0.010 | | 0.508 |
| Palatal.alveolar |  | -0.031 | | 2.062 | -4.073 | 4.011 | -0.15 | 0.010 | | 0.508 |
| Glottal |  | 2.790 | | 1.628 | -0.401 | 5.981 | 1.71 | 0.010 | | 0.508 |
| velar |  | 3.455 | | 1.608 | 0.303 | 6.607 | 2.15 | 0.010 | | 0.508 |
| voiced |  | -0.027 | | 0.775 | -1.546 | 1.492 | -0.03 | 0.010 | | 0.508 |
|  |  |  | |  |  |  |  |  | |  |
| *Experiment 3a: Unspaced compound word- Identification of the Modifier lexeme ^a^* |  |  | |  |  |  |  |  | |  |
| L |  | -0.150 | | 0.377 | -0.889 | 0.589 | -0.40 | 0.001 | | 0.410 |
| CFreq |  | 0.616 | | 0.361 | -0.092 | 1.324 | 1.71 | 0.001 | | 0.410 |
| MFreq |  | -0.278 | | 0.348 | -0.960 | 0.404 | -0.80 | 0.001 | | 0.410 |
| HFreq |  | **-0.804** | | **0.349** | **-1.488** | **-0.120** | **-2.31*** | **0.001** | | **0.410** |
| bilabial |  | -0.467 | | 0.648 | -1.737 | 0.803 | -0.72 | 0.001 | | 0.410 |
| labiodental |  | -1.469 | | 0.824 | -3.084 | 0.146 | -1.78 | 0.001 | | 0.410 |
| dental |  | -2.084 | | 2.144 | -6.286 | 2.118 | -0.97 | 0.001 | | 0.410 |
| labiovelar |  | -0.838 | | 0.753 | -2.314 | 0.638 | -1.11 | 0.001 | | 0.410 |
| alveolar |  | -0.460 | | 0.676 | -1.785 | 0.865 | -0.68 | 0.001 | | 0.410 |
| Palatal.alveolar |  | 0.774 | | 1.054 | -1.292 | 2.840 | 0.73 | 0.001 | | 0.410 |
| Glottal |  | -0.465 | | 0.857 | -2.145 | 1.215 | 0.54 | 0.001 | | 0.410 |
| velar |  | -0.808 | | 0.858 | -2.490 | 0.874 | -0.94 | 0.001 | | 0.410 |
| voiced |  | -0.517 | | 0.408 | -1.317 | 0.283 | -1.27 | 0.001 | | 0.410 |
| *Experiment 3a: Unspaced compound word- Identification of the Head lexeme ^a^* |  |  | |  |  |  |  |  | |  |
| L |  | 0.165 | | 0.390 | -0.599 | 0.929 | 0.42 | 0.001 | | 0.373 |
| CFreq |  | 0.072 | | 0.382 | -0.677 | 0.821 | 0.19 | 0.001 | | 0.373 |
| MFreq |  | -0.516 | | 0.361 | -1.224 | 0.192 | 1.43 | 0.001 | | 0.373 |
| HFreq |  | 0.257 | | 0.356 | -0.441 | 0.955 | 0.72 | 0.001 | | 0.373 |
| bilabial |  | -0.420 | | 0.663 | -1.719 | 0.879 | -0.63 | 0.001 | | 0.373 |
| labiodental |  | 0.700 | | 0.857 | -0.980 | 2.380 | 0.82 | 0.001 | | 0.373 |
| dental |  | 0.875 | | 2.071 | -3.184 | 4.934 | 0.42 | 0.001 | | 0.373 |
| labiovelar |  | -0.280 | | 0.775 | -1.799 | 1.239 | -0.36 | 0.001 | | 0.373 |
| alveolar |  | -0.854 | | 0.696 | -2.218 | 0.510 | -1.23 | 0.001 | | 0.373 |
| Palatal.alveolar |  | -0.144 | | 1.174 | -2.445 | 2.157 | -0.12 | 0.001 | | 0.373 |
| Glottal |  | -0.136 | | 0.886 | -1.873 | 1.601 | -0.15 | 0.001 | | 0.373 |
| velar |  | 0.525 | | 0.866 | -1.172 | 2.222 | 0.61 | 0.001 | | 0.373 |
| voiced |  | 0.143 | | 0.423 | -0.686 | 0.972 | 0.34 | 0.001 | | 0.373 |
| *Experiment 3b: Spaced compound word –Identification of the Modifier lexeme ^a^* |  |  | |  |  |  |  |  | |  |
| L |  | 1.436 | | 0.645 | 0.172 | 2.700 | 2.23 | 0.012 | | 0.411 |
| CFreq |  | 0.055 | | 0.625 | -1.170 | 1.280 | 0.88 | 0.012 | | 0.411 |
| MFreq |  | **2.252** | | **0.596** | **1.084** | **3.420** | **3.78*** | **0.012** | | **0.411** |
| HFreq |  | -0.577 | | 0.593 | -1.739 | 0.585 | -0.97 | 0.012 | | 0.411 |
| bilabial |  | 0.997 | | 1.104 | -1.167 | 3.161 | 0.90 | 0.012 | | 0.411 |
| labiodental |  | 0.050 | | 1.415 | -2.723 | 2.823 | 0.04 | 0.012 | | 0.411 |
| dental |  | 5.099 | | 3.577 | -1.912 | 12.11 | 1.43 | 0.012 | | 0.411 |
| labiovelar |  | -0.145 | | 1.289 | -2.671 | 2.381 | -0.11 | 0.012 | | 0.411 |
| alveolar |  | -1.477 | | 1.157 | -3.745 | 0.791 | -1.28 | 0.012 | | 0.411 |
| Palatal.alveolar |  | -2.573 | | 1.862 | -6.223 | 1.077 | -1.38 | 0.012 | | 0.411 |
| Glottal |  | 0.261 | | 1.470 | -2.620 | 3.142 | 0.18 | 0.012 | | 0.411 |
| velar |  | 0.980 | | 1.452 | -1.866 | 3.826 | 0.68 | 0.012 | | 0.411 |
| voiced |  | -0.801 | | 0.699 | -2.171 | 0.569 | -1.15 | 0.012 | | 0.411 |
| *Experiment 3b: Spaced compound word - Identification of the Head lexeme ^a^* |  |  | |  |  |  |  |  | |  |
| L |  | 0.544 | | 0.652 | -10.40 | 14.49 | 0.84 | 0.011 | | 0.382 |
| CFreq |  | -0.540 | | 0.632 | -15.56 | 8.627 | -0.86 | 0.011 | | 0.382 |
| MFreq |  | -0.460 | | 0.601 | -11.97 | 11.93 | -0.77 | 0.011 | | 0.382 |
| HFreq |  | **1.947** | | **0.598** | **-11.52** | **11.65** | **3.25*** | **0.011** | | **0.382** |
| bilabial |  | 2.636 | | 1.114 | -27.77 | 14.77 | 2.37 | 0.011 | | 0.382 |
| labiodental |  | 0.981 | | 1.428 | -3.096 | 51.16 | 0.69 | 0.011 | | 0.382 |
| dental |  | 7.857 | | 3.601 | -102.1 | 39.25 | 2.18 | 0.011 | | 0.382 |
| labiovelar |  | **3.454** | | **1.301** | **-23.80** | **25.79** | **2.65*** | **0.011** | | **0.382** |
| alveolar |  | 1.353 | | 1.167 | -7.625 | 36.95 | 1.16 | 0.011 | | 0.382 |
| Palatal.alveolar |  | -0.652 | | 1.878 | -56.17 | 14.55 | -0.35 | 0.011 | | 0.382 |
| Glottal |  | **3.914** | | **1.484** | **-25.77** | **31.66** | **2.64*** | **0.011** | | **0.382** |
| velar |  | **4.352** | | **1.465** | **-24.84** | **31.37** | **2.97*** | **0.011** | | **0.382** |
| voiced |  | -0.058 | | 0.705 | -30.76 | 3.384 | -0.08 | 0.011 | | 0.382 |

|  |
| --- |
|  |

*Note***.** orthographic length (L) and frequency (CFreq) of the compound word, word frequency of the modifier (MFreq) and head (HFreq). SE = Standard error. *Significant at the *p* = .05 level after Holm-Bonferroni method and bolded. ^a^ This model converged only with a random subject and item intercept and no predictor was included as part of a random slope.

**Table S5.**

*The baseline model + predictors for stimulus duration of correct responses in the progressive demasking task with phonetic complexity added as control variables.*

| Effect |  |  | Stimulus duration | | | | | |
| --- | --- | --- | --- | --- | --- | --- | --- | --- |
|  | *Β* | | *SE* | *2.5%* | *97.5%* | *T* | *R2m* | *R2c* |
| *Experiment 1: Unspaced compound word PDT^a^* |  | |  |  |  |  |  |  |
| CFam | **-3.652** | | **1.166** | **-5.937** | **-1.367** | **-3.13*** | **0.085** | **0.418** |
| CAoA | **7.618** | | **1.202** | **5.262** | **9.974** | **6.34*** | **0.105** | **0.416** |
| CI | **7.876** | | **1.111** | **5.698** | **10.054** | **-7.09*** | **0.110** | **0.416** |
| ST | -1.401 | | 1.183 | -3.720 | 0.918 | -1.19 | 0.078 | 0.418 |
| LMD | -2.243 | | 1.220 | -4.634 | 0.148 | -1.84 | 0.080 | 0.418 |
| NA (H) | **4.002** | | **1.174** | **1.701** | **6.303** | **-3.41*** | **0.087** | **0.418** |
| NA (%) | **-4.161** | | **1.170** | **-6.454** | **-1.868** | **-3.56*** | **0.088** | **0.418** |
| IA | -1.516 | | 1.181 | -3.831 | 0.799 | -1.28 | 0.079 | 0.418 |
| VC | 1.552 | | 1.188 | -0.776 | 3.880 | 1.31 | 0.079 | 0.418 |
| MFam | 1.828 | | 1.605 | -1.318 | 4.974 | 1.14 | 0.078 | 0.418 |
| MAoA | **4.562** | | **1.406** | **1.806** | **7.318** | **3.24*** | **0.086** | **0.418** |
| MI | -2.974 | | 1.168 | -5.263 | -0.685 | -2.55 | 0.082 | 0.418 |
| Hfam | -1.658 | | 1.485 | -4.569 | 1.253 | -1.12 | 0.078 | 0.418 |
| HAoA | 2.649 | | 1.375 | -0.046 | 5.344 | 1.93 | 0.080 | 0.418 |
| HI | -2.744 | | 1.200 | -5.096 | -0.392 | -2.29 | 0.081 | 0.418 |
|  |  | |  |  |  |  |  |  |
| *Experiment 2: Spaced compound word PDT^b,c^* |  | |  |  |  |  |  |  |
| CFam | **-2.318** | | **0.628** | **-3.549** | **-1.087** | **-3.69*** | **0.014** | **0.509** |
| CAoA | **5.428** | | **0.582** | **4.287** | **6.569** | **9.33*** | **0.028** | **0.508** |
| CI | **-4.987** | | **0.565** | **-6.094** | **-3.880** | **-8.82*** | **0.026** | **0.508** |
| ST | 0.947 | | 0.671 | -0.368 | 2.262 | -1.41 | 0.010 | 0.509 |
| LMD | -1.564 | | 0.635 | -2.809 | -0.319 | -2.46 | 0.012 | 0.509 |
| NA (H) | **1.917** | | **0.647** | **0.649** | **3.185** | **2.96*** | **0.012** | **0.509** |
| NA (%) | **-2.368** | | **0.636** | **-3.615** | **-1.121** | **-3.73*** | **0.014** | **0.509** |
| IA | -1.534 | | 0.635 | -2.779 | -0.289 | -2.42 | 0.012 | 0.509 |
| VC | 1.604 | | 0.638 | 0.354 | 2.854 | 2.51 | 0.012 | 0.509 |
| MFam | 0.590 | | 0.879 | -1.133 | 2.313 | 0.67 | 0.010 | 0.509 |
| MAoA | **2.756** | | **0.760** | **1.266** | **4.246** | **3.63*** | **0.014** | **0.509** |
| MI | **-1.839** | | **0.634** | **-3.082** | **-0.596** | **-2.90*** | **0.012** | **0.509** |
| Hfam | -1.002 | | 0.809 | -2.588 | 0.584 | -1.24 | 0.010 | 0.509 |
| HAoA | **2.059** | | **0.739** | **0.611** | **3.507** | **2.79*** | **0.012** | **0.509** |
| HI | -1.524 | | 0.654 | -2.806 | -0.242 | -2.33 | 0.012 | 0.509 |
|  |  | |  |  |  |  |  |  |
| *Experiment 3a: Unspaced compound word- Identification of the Modifier lexeme ^a^* |  | |  |  |  |  |  |  |
| CFam | **-0.872** | | **0.350** | **-1.558** | **-0.186** | **-2.49*** | **0.002** | **0.410** |
| CAoA | 0.567 | | 0.396 | -0.209 | 1.343 | 1.43 | 0.001 | 0.410 |
| CI | -0.005 | | 0.375 | -0.74 | 0.730 | -0.01 | 0.001 | 0.410 |
| ST | 0.302 | | 0.356 | -0.396 | 0.998 | 0.85 | 0.001 | 0.410 |
| LMD | -0.198 | | 0.336 | -0.857 | 0.461 | -0.59 | 0.001 | 0.410 |
| NA (H) | 0.630 | | 0.351 | -0.058 | 1.318 | 1.79 | 0.002 | 0.410 |
| NA (%) | 0.770 | | 0.349 | 0.086 | 1.454 | -2.21 | 0.002 | 0.410 |
| IA | -0.478 | | 0.342 | -1.148 | 0.192 | -1.40 | 0.001 | 0.410 |
| VC | -0.144 | | 0.034 | -0.211 | -0.077 | -0.42 | 0.001 | 0.410 |
| MFam | -0.014 | | 0.461 | -0.918 | 0.890 | -0.03 | 0.001 | 0.410 |
| MAoA | -0.396 | | 0.417 | -1.213 | 0.421 | -0.95 | 0.001 | 0.410 |
| MI | -0.067 | | 0.340 | -0.733 | 0.599 | -0.20 | 0.001 | 0.410 |
| Hfam | -0.713 | | 0.423 | -1.542 | 0.116 | -1.69 | 0.001 | 0.410 |
| HAoA | 0.049 | | 0.396 | -0.727 | 0.825 | 0.12 | 0.001 | 0.410 |
| HI | -0.461 | | 0.349 | -1.145 | 0.223 | -1.32 | 0.001 | 0.410 |
|  |  | |  |  |  |  |  |  |
| *Experiment 3a: Unspaced compound word- Identification of the Head lexeme ^a^* |  | |  |  |  |  |  |  |
| CFam | 0.463 | | 0.355 | -0.233 | 1.159 | 1.30 | 0.001 | 0.373 |
| CAoA | -0.712 | | 0.403 | -1.502 | 0.078 | -1.76 | 0.001 | 0.373 |
| CI | 0.701 | | 0.384 | -0.052 | 1.454 | 1.83 | 0.001 | 0.374 |
| ST | 0.539 | | 0.366 | -0.178 | 1.256 | 1.48 | 0.001 | 0.373 |
| LMD | -0.042 | | 0.357 | -0.742 | 0.658 | -0.12 | 0.001 | 0.373 |
| NA (H) | -0.056 | | 0.362 | -0.766 | 0.654 | -0.15 | 0.001 | 0.373 |
| NA (%) | -0.187 | | 0.367 | -0.906 | 0.532 | -0.51 | 0.001 | 0.373 |
| IA | -0.146 | | 0.354 | -0.84 | 0.548 | -0.41 | 0.001 | 0.373 |
| VC | -0.475 | | 0.354 | -1.169 | 0.219 | -1.34 | 0.001 | 0.373 |
| MFam | 0.474 | | 0.479 | -0.465 | 1.413 | 0.99 | 0.001 | 0.373 |
| MAoA | -0.273 | | 0.433 | -1.122 | 0.576 | -0.63 | 0.001 | 0.373 |
| MI | 0.486 | | 0.359 | -0.218 | 1.190 | 1.36 | 0.001 | 0.373 |
| Hfam | 0.038 | | 0.446 | -0.836 | 0.912 | 0.09 | 0.001 | 0.373 |
| HAoA | -0.301 | | 0.418 | -1.12 | 0.518 | -0.72 | 0.001 | 0.373 |
| HI | 0.226 | | 0.365 | -0.489 | 0.941 | 0.62 | 0.001 | 0.373 |
|  |  | |  |  |  |  |  |  |
| *Experiment 3b: Spaced compound word –Identification of the Modifier lexeme ^a^* |  | |  |  |  |  |  |  |
| CFam | -1.292 | | 0.584 | -2.437 | -0.147 | -2.21 | 0.013 | 0.410 |
| CAoA | **2.974** | | **0.624** | **1.751** | **4.197** | **4.76*** | **0.018** | **0.410** |
| CI | **-3.326** | | **0.573** | **-4.449** | **-2.203** | **-5.80*** | **0.020** | **0.410** |
| ST | -0.784 | | 0.606 | -1.972 | 0.404 | -1.29 | 0.012 | 0.410 |
| LMD | -1.082 | | 0.578 | -2.215 | 0.051 | -1.87 | 0.013 | 0.410 |
| NA (H) | **1.947** | | **0.579** | **0.812** | **3.082** | **3.36*** | **0.015** | **0.410** |
| NA (%) | **-2.359** | | **0.567** | **-3.47** | **-1.248** | **-4.16*** | **0.017** | **0.410** |
| IA | -1.307 | | 0.576 | -2.436 | -0.178 | -2.27 | 0.013 | 0.410 |
| VC | 0.597 | | 0.586 | -0.552 | 1.746 | 1.02 | 0.012 | 0.410 |
| MFam | 1.669 | | 0.781 | 0.138 | 3.2 | 2.14 | 0.013 | 0.410 |
| MAoA | 0.490 | | 0.717 | -0.915 | 1.895 | 0.68 | 0.012 | 0.410 |
| MI | -0.157 | | 0.589 | -1.311 | 0.997 | -0.27 | 0.012 | 0.410 |
| Hfam | -0.566 | | 0.732 | -2.001 | 0.869 | -0.77 | 0.012 | 0.410 |
| HAoA | 1.591 | | 0.672 | 0.274 | 2.908 | 2.37 | 0.013 | 0.410 |
| HI | **-2.132** | | **0.573** | **-3.255** | **-1.009** | **-3.72*** | **0.016** | **0.410** |
|  |  | |  |  |  |  |  |  |
| *Experiment 3b: Spaced compound word –Identification of the Head lexeme ^a^* |  | |  |  |  |  |  |  |
| CFam | 0.799 | | 0.597 | -0.371 | 1.969 | -1.34 | 0.012 | 0.383 |
| CAoA | **3.495** | | **0.611** | **2.297** | **4.693** | **5.72*** | **0.020** | **0.382** |
| CI | **3.674** | | **0.564** | **2.569** | **4.779** | **-6.51*** | **0.021** | **0.383** |
| ST | **-1.598** | | **0.601** | **-2.776** | **-0.420** | **-2.66*** | **0.013** | **0.383** |
| LMD | -1.166 | | 0.583 | -2.309 | -0.023 | -2.00 | 0.013 | 0.383 |
| NA (H) | **1.691** | | **0.591** | **0.533** | **2.849** | **2.86*** | **0.014** | **0.383** |
| NA (%) | **-1.588** | | **0.594** | **-2.752** | **-0.424** | **-2.67*** | **0.013** | **0.383** |
| IA | -0.959 | | 0.586 | -2.108 | 0.190 | -1.64 | 0.012 | 0.383 |
| VC | 0.477 | | 0.592 | -0.683 | 1.637 | 0.805 | 0.012 | 0.383 |
| MFam | 0.907 | | 0.798 | -0.657 | 2.471 | 1.14 | 0.012 | 0.383 |
| MAoA | 1.700 | | 0.710 | 0.308 | 3.092 | 2.39 | 0.013 | 0.383 |
| MI | -1.374 | | 0.583 | -2.517 | -0.231 | -2.36 | 0.013 | 0.383 |
| Hfam | -0.638 | | 0.739 | -2.086 | 0.810 | -0.86 | 0.012 | 0.383 |
| HAoA | 0.577 | | 0.691 | -0.777 | 1.931 | 0.83 | 0.012 | 0.383 |
| HI | -0.872 | | 0.602 | -2.052 | 0.308 | -1.45 | 0.012 | 0.383 |

*Note***.** Conceptual familiarity of the compound word (CFam), age of acquisition of the compound word (CAoA), imageability of the compound word (CI), semantic transparency (ST), Lexeme meaning dominance (LMD), name agreement: H index (NA(H)) and percent (%), image agreement (IA) visual complexity (VC) of the compound word, familiarity of the modifier lexeme (MFam), Age of acquisition of the modifier lexeme (MAoA) and imageability of the modifier lexeme (MI). Familiarity of the head lexeme (Hfam), age of acquisition of the head lexeme (HAoA) and imageability of the head lexeme (HI). SE = Standard error. *Significant at the *p* = .05 level after Holm-Bonferroni method and bolded. ^a^ This model converged only with a random subject and item intercept and no predictor was included as part of a random slope ^b^ This model only converged for stimulus duration of correct responses when a random subject-intercept model is included ^c^ This model only converged for accuracy when a random subject-intercept model is included. Each predictor was added individually to the baseline model.

**Table 4.**

*The baseline model results for accuracy and stimulus duration of correct responses in the visual duration threshold task with phonetic complexity included in the model*

| Effect |  |  | Accuracy | | | | | | |  | Stimulus duration | | | |  |  |
| --- | --- | --- | --- | --- | --- | --- | --- | --- | --- | --- | --- | --- | --- | --- | --- | --- |
|  | *β* | | *SE* | *2.5%* | *97.5%* | *T* | *R2m* | *R2c* |  | *β* | *SE* | *2.5%* | *97.5%* | *T* | *R2m* | *R2c* |
| *Visual Duration Threshold^a^* |  | |  |  |  |  |  |  |  |  |  |  |  |  |  |  |
| L | 0.727 | | 2.221 | -3.626 | 5.080 | 0.33 | 0.103 | 0.592 |  | 0.291 | 0.905 | -1.483 | 2.065 | 0.32 | 0.042 | 0.433 |
| CFreq | 0.144 | | 0.198 | -0.244 | 0.532 | 0.08 | 0.103 | 0.592 |  | -1.934 | 0.878 | -3.655 | -0.213 | -2.20 | 0.042 | 0.433 |
| MFreq | **0.081** | | **0.179** | **-0.270** | **0.432** | **3.05*** | **0.103** | **0.592** |  | -0.264 | 0.836 | -1.903 | 1.375 | 0.32 | 0.042 | 0.433 |
| HFreq | 0.508 | | 0.187 | 0.141 | 0.875 | 0.07 | 0.103 | 0.592 |  | **-1.981** | **0.829** | **-3.606** | **-0.356** | **-2.39*** | **0.042** | **0.433** |
| bilabial | -0.451 | | 0.345 | -1.127 | 0.225 | -1.07 | 0.103 | 0.592 |  | 2.175 | 1.512 | -0.789 | 5.139 | 1.44 | 0.042 | 0.433 |
| labiodental | -0.306 | | 0.444 | -1.176 | 0.564 | 0.21 | 0.103 | 0.592 |  | 0.339 | 1.968 | -3.518 | 4.196 | 0.17 | 0.042 | 0.433 |
| dental | 0.398 | | 1.003 | -1.568 | 2.364 | -1.06 | 0.103 | 0.592 |  | -0.265 | 5.029 | -10.12 | 9.592 | -0.05 | 0.042 | 0.433 |
| labiovelar | -0.329 | | 0.397 | -1.107 | 0.449 | -1.35 | 0.103 | 0.592 |  | 3.093 | 1.790 | -0.415 | 6.601 | 1.73 | 0.042 | 0.433 |
| alveolar | -0.468 | | 0.367 | -1.187 | 0.251 | -0.19 | 0.103 | 0.592 |  | 0.830 | 0.830 | -0.797 | 2.457 | 0.52 | 0.042 | 0.433 |
| Palatal.alveolar | -0.548 | | -0.510 | 0.452 | -1.548 | -0.88 | 0.103 | 0.592 |  | 3.489 | 2.601 | -1.609 | 8.587 | 1.34 | 0.042 | 0.433 |
| Glottal | -0.709 | | -0.074 | -0.564 | -0.854 | -0.16 | 0.103 | 0.592 |  | 2.424 | 2.048 | -1.590 | 6.438 | 1.18 | 0.042 | 0.433 |
| velar | -0.430 | | 0.448 | -1.308 | 0.448 | -1.29 | 0.103 | 0.592 |  | 2.431 | 2.024 | -1.536 | 6.398 | 1.20 | 0.042 | 0.433 |
| voiced | -0.064 | | 0.217 | -0.489 | 0.361 | 0.45 | 0.103 | 0.592 |  | 0.198 | 0.980 | -1.723 | 2.119 | 0.20 | 0.042 | 0.433 |

|  |
| --- |
|  |

*Note***.** orthographic length (L) and frequency (CFreq) of the compound word, word frequency of the modifier lexeme (MFreq) and word frequency of the head llexeme (HFreq). SE = Standard error. *Significant at the *p* = .05 level after Holm-Bonferroni method and bolded. ^a^ This model converged only with a random subject and item intercept and no predictor was included as part of a random slope ^b^ This model only converged for stimulus duration of correct responses when a random subject-intercept model is included ^c^ This model only converged for accuracy when a random subject-intercept model is included.

**Table S7.**

*The baseline + predictors model results for accuracy and stimulus duration of correct responses in the visual duration threshold task with phonetic complexity included in the model.*

| Effect |  |  | Accuracy | | | | | |  | | Stimulus duration | | |  | |  | |
| --- | --- | --- | --- | --- | --- | --- | --- | --- | --- | --- | --- | --- | --- | --- | --- | --- | --- |
|  | *Β* | | *SE* | *2.5%* | *97.5%* | *T* | *R2m* | *R2c* |  | *β* | *SE* | *2.5%* | *97.5%* | *T* | *R2m* | | *R2c* |
| *Visual Duration Threshold^a^* |  | |  |  |  |  |  |  |  |  |  |  |  |  |  | |  |
| CFam | **0.723** | | **0.178** | **0.374** | **1.072** | **4.07*** | **0.088** | **0.334** |  | -1.793 | 0.817 | -3.394 | -0.192 | -2.19 | 0.051 | | 0.431 |
| CAoA | **-1.538** | | **0.167** | **-1.865** | **-1.211** | **-9.21*** | **0.165** | **0.323** |  | **5.694** | **0.810** | **4.106** | **7.282** | **7.03*** | **0.107** | | **0.424** |
| CI | **1.221** | | **0.150** | **0.927** | **1.515** | **8.14*** | **0.135** | **0.314** |  | **-4.281** | **0.807** | **-5.863** | **-2.699** | **-5.97*** | **0.086** | | **0.422** |
| ST | 0.357 | | 0.187 | -0.010 | 0.7235 | 1.91 | 0.064 | 0.332 |  | -0.857 | 0.848 | -2.519 | 0.8051 | -1.01 | 0.043 | | 0.434 |
| LMD | 0.364 | | 0.182 | 0.007 | 0.721 | 2.00 | 0.067 | 0.335 |  | -0.951 | 0.816 | -2.550 | 0.648 | -1.17 | 0.045 | | 0.434 |
| NA (H) | **-1.002** | | **0.166** | **-1.327** | **-0.677** | **-6.02*** | **0.117** | **0.332** |  | **2.762** | **0.808** | **1.178** | **4.346** | **3.42*** | **0.062** | | **0.431** |
| NA (%) | **0.961** | | **0.167** | **0.634** | **1.288** | **5.75*** | **0.111** | **0.332** |  | **-2.867** | **0.806** | **-4.447** | **-1.287** | **-3.56*** | **0.062** | | **0.431** |
| IA | **0.933** | | **0.173** | **0.594** | **1.272** | **5.41*** | **0.111** | **0.340** |  | **-3.748** | **0.748** | **-5.214** | **-2.282** | **-5.01*** | **0.083** | | **0.431** |
| VC | -0.456 | | 0.176 | -0.801 | -0.111 | -2.59 | 0.071 | 0.331 |  | **2.463** | **0.800** | **0.895** | **4.031** | **3.08*** | **0.058** | | **0.431** |
| MFam | -0.190 | | 0.251 | -0.682 | 0.302 | -0.76 | 0.060 | 0.336 |  | 0.669 | 1.113 | -1.512 | 2.850 | 0.60 | 0.043 | | 0.431 |
| MAoA | **-0.739** | | **0.208** | **-1.147** | **-0.331** | **-3.55*** | **0.079** | **0.331** |  | **3.675** | **0.962** | **1.789** | **5.561** | **3.82*** | **0.065** | | **0.431** |
| MI | **0.535** | | **0.173** | **0.196** | **0.874** | **3.09*** | **0.076** | **0.336** |  | **-2.327** | **0.802** | **-3.899** | **-0.755** | **-2.90*** | **0.057** | | **0.433** |
| Hfam | 0.362 | | 0.223 | -0.075 | 0.799 | 1.62 | 0.064 | 0.335 |  | -0.562 | 1.031 | -2.583 | 1.459 | -0.55 | 0.043 | | 0.431 |
| HAoA | -0.552 | | 0.211 | -0.966 | -0.138 | -2.62 | 0.071 | 0.331 |  | 2.301 | 0.939 | 0.461 | 4.141 | 2.45 | 0.052 | | 0.431 |
| HI | **0.550** | | **0.178** | **0.201** | **0.899** | **3.09*** | **0.076** | **0.335** |  | **-2.502** | **0.817** | **-4.103** | **-0.901** | **-3.07*** | **0.058** | | **0.431** |

|  |
| --- |

*Note***.** Conceptual familiarity of the compound word (CFam), age of acquisition of the compound word (CAoA), imageability of the compound word (CI), semantic transparency (ST), Lexeme meaning dominance (LMD), name agreement: H index (NA(H)) and percent (%), image agreement (IA) visual complexity (VC) of the compound word, familiarity of the modifier lexeme (MFam), Age of Acquisition of the modifier lexeme (MAoA) and imageability of the modifier (MI). Familiarity of the head lexeme (Hfam), Age of Acquisition of the head lexeme (HAoA) and imageability of the head lexeme (HI). SE = Standard error. *Significant at the *p* = .05 level after Holm-Bonferroni method and bolded. ^a^ This model converged only with a random subject and item intercept and no predictor was included as part of a random slope ^b^ This model only converged for stimulus duration of correct responses when a random subject-intercept model is included ^c^ This model only converged for accuracy when a random subject-intercept model is included.
